# Methylated lncRNAs suppress apoptosis of gastric cancer stem cells via the lncRNA–miRNA/protein axis

**DOI:** 10.1186/s11658-024-00568-8

**Published:** 2024-04-10

**Authors:** Yuan Ci, Yuan Zhang, Xiaobo Zhang

**Affiliations:** College of Life Sciences, Laboratory for Marine Biology and Biotechnology of Pilot National Laboratory for Marine Science and Technology (Qingdao), Southern Marine Science and Engineering Guangdong Laboratory (Zhuhai), Zhejiang University, Hangzhou, 310058 People’s Republic of China

**Keywords:** Gastric cancer stem cells, LncRNA, Methylation, Apoptosis, Stemness

## Abstract

**Background:**

Long noncoding RNAs (lncRNAs) play essential roles in the tumorigenesis of gastric cancer. However, the influence of lncRNA methylation on gastric cancer stem cells (GCSCs) remains unclear.

**Methods:**

The N6-methyladenosine (m6A) levels of lncRNAs in gastric cancer stem cells were detected by methylated RNA immunoprecipitation sequencing (MeRIP-seq), and the results were validated by MeRIP-quantitative polymerase chain reaction (qPCR). Specific sites of m6A modification on lncRNAs were detected by single-base elongation- and ligation-based qPCR amplification (SELECT). By constructing and transfecting the plasmid expressing methyltransferase-like 3 (METTL3) fused with catalytically inactivated Cas13 (dCas13b) and guide RNA targeting specific methylation sites of lncRNAs, we obtained gastric cancer stem cells with site-specific methylation of lncRNAs. Reverse transcription (RT)-qPCR and Western blot were used for detecting the stemness of treated gastric cancer stem cells.

**Results:**

The site-specific methylation of *PSMA3-AS1* and *MIR22HG* suppressed apoptosis and promoted stemness of GCSCs. LncRNA methylation enhanced the stability of *PSMA3-AS1* and *MIR22HG* to suppress apoptosis of GCSCs via the *PSMA3-AS1*–miR-411-3p– or *MIR22HG*–miR-24-3p–*SERTAD1* axis. Simultaneously, the methylated lncRNAs promoted the interaction between *PSMA3-AS1* and the EEF1A1 protein or *MIR22HG* and the LRPPRC protein, stabilizing the proteins and leading to the suppression of apoptosis. The in vivo data revealed that the methylated *PSMA3-AS1* and *MIR22HG* triggered tumorigenesis of GCSCs.

**Conclusions:**

Our study revealed the requirement for site-specific methylation of lncRNAs in the tumorigenesis of GCSCs, contributing novel insights into cancer development.

**Supplementary Information:**

The online version contains supplementary material available at 10.1186/s11658-024-00568-8.

## Background

Cancer has been a hot topic in biological investigations for decades. Regarding the origin of cancer, two hypotheses have been proposed [1]. The gene mutation theory posits that the accumulation of mutations in cancer-related genes triggers tumorigenesis [2, 3]. Another hypothesis, which suggests that tumorigenesis results from the infinite proliferation of cancer stem cells, has become the dominant doctrine in recent years [4]. Cancer stem cells (CSCs), a subclass of neoplastic cells within tumors, possess the ability to self-renew and undergo aberrant differentiation [5]. Over the past decades, there has been an increasing interest in cancer stem cells, because CSCs are not only responsible for tumor initiation, propagation, and maintenance, but also contribute to chemotherapy resistance and relapse of tumors [5, 6]. Therefore, exploring the factors that affect the biological activities especially the differentiation of CSCs becomes urgently required. It is found that some signaling pathways, such as the Hedgehog, Notch, Wnt/β-catenin, and NF-κB, contribute to the enhancement of the self-renewal and differentiation abilities of CSCs [7]. The dysregulation of the genes of these signaling pathways has great effects on cancer stem cells. Long noncoding RNAs (lncRNAs), acting as regulatory factors in gene expression, play pivotal roles in the cellular activities of CSCs [8–10]. However, the epigenetic regulatory mechanisms of RNAs, especially lncRNAs, have not been extensively explored.

In recent years, RNA modification, one of the epigenetic regulatory mechanisms of RNAs, has attracted more and more attention [11, 12]. It is found that N6-methyladenosine (m6A) reversible modification plays essential roles in post-transcriptional and translational regulation [13]. In mammals, the formation and removal of m6A of RNA occurs by the m6A methyltransferases called “writers” and demethylases called “erasers” during transcription, respectively, which rely on the m6A-binding proteins called “readers” in either nucleus or cytoplasm [14]. Generally, most m6A modification of mRNAs is installed by m6A methyltransferase complex composed of METTL3, METTL14, and WTAP [15]. METTL16 and METTL5 may function as the m6A “writers” of some noncoding RNAs [16, 17]. Both alkylation repair homolog protein 5 (ALKBH5) and fat-mass and obesity-associated protein (FTO) are well-documented m6A “erasers” [18, 19]. The “readers” of m6A RNA mainly include YTH (YT521-B homology) domain-containing family protein 1 (YTHDF1), YTHDF2, YTHDF3, YTH domain-containing protein 1 (YTHDC1), YTHDC2, and insulin-like growth factor 2 mRNA-binding proteins (IGF2BPs) [20, 21]. As reported, m6A modification has significant effects on the stemness maintenance and differentiation of stem cells and cancer stem cells [22–24]. However, the m6A modification of lncRNAs and its impact on CSCs remain unexplored.

To address this issue, the differences of lncRNA m6A modification between gastric cancer stem cells (GCSCs) and gastric cancer nonstem cells (GCNSCs), which were isolated from patients with gastric cancer (GC), was characterized in this study. The findings unveiled that the site-specific m6A modification of lncRNAs *PSMA3-AS1* and *MIR22HG* promotes tumorigenesis in GCSCs by suppressing apoptosis and promoting proliferation through increased stability of miRNAs and proteins..

## Methods

### Sorting and culture of GCSCs and GCNSCs

GCSCs were sorted from the solid tumors of a patient with GC. The solid tumors were washed by phosphate buffered saline (PBS) and were subsequently sliced into 1 mm^3^ fragments. Following this, they were subjected to a 5 h digestion using collagenase I (Gibco). The mixture was filtrated through a 200 mesh filter and then centrifuged at 100 × g for 10 min. Then the cell suspension was cultured in serum-free Dulbecco’s modified Eagle's medium (DMEM)/F12 medium (Gibco) containing 2% B27 (Invitrogen), 5 μg/ml insulin (Sigma), 20 ng/ml basic fibroblast growth factor (bFGF; FSigma), 20 ng/ml epidermal growth factor (EGF; Sigma), and 1% penicillin–streptomycin solution (Gibco). After culture for 1 week, the cells derived from tumorspheres were identified as GCSCs. Tumorsphere formation assay and western blotting for stemness gene were used for identification of the separated GCSCs. The residual cells, which failed to form tumorspheres were regarded as GCNSCs and cultured in DMEM basic medium (Gibco) with 10% fetal bovine serum (FBS; Gibco) and 1% penicillin–streptomycin solution (Gibco).

Isolation of stem cells from the GC cell lines HGC-27 and MGC-803 has been described in our previous report [25]. Briefly, ALDEFLUOR Kit (Cyagen) was employed to isolate GCSCs, which was based on the expression of ALDH1, a recognized marker for GCSCs. Cells suspended in buffer with ALDH1 fluorescent substrate BODIPY-aminoacetate (BAAA, 1 μmol/L) or diethylaminobenzaldehyde (DEAB), a specific inhibitor of ALDH1, were subjected to fluorescence activated cell sorting (FACS).

### Western blot

Proteins was released using lysis buffer with protease inhibitors, and were then loaded onto sodium dodecyl-sulfate polyacrylamide gel electrophoresis (SDS-PAGE) gels for separation. Next, the proteins were transferred onto a nitrocellulose membrane, which was successively blocked with 5% nonfat milk. Incubation with primary antibodies (Abcam) against the target proteins overnight at 4 °C, followed by washing and incubation with horseradish peroxidase (HRP)-conjugated secondary antibodies (Jackson ImmunoResearch). The chemiluminescence signal was examined using ECL kit (PerkinElmer).

### Tumorsphere formation assay

A single cell was cultured in a 96-well ultra-low attachment microplate (Corning) for 15 days, and the status of cells was recorded by light microscope. At various time points after culture, the percentage of tumorsphere formation was calculated.

### Methylated RNA immunoprecipitation sequencing (MeRIP-seq)

After removal of rRNAs by RiboMinus™ Eukaryote Kit v2 (Invitrogen), total RNAs were interrupted into ~ 100 nt by RNA fragmentation reagents (Invitrogen). Subsequently the RNAs were incubated with anti-m6A antibody (Abcam) and Protein A/G Magnetic Beads (Thermo Scientific) at 4 ℃ overnight. The RNA fragments bound with beads were eluted and subjected to RNA sequencing or reverse transcription-quantitative polymerase chain reaction (RT-qPCR).

### Transwell migration assay

Cell migration was assessed using a Transwell^®^ 24-well Permeable Support (Corning, USA). Cells were seeded in serum-free medium and cultured for 24 h. Then, 5 × 10^4^ cells were placed into the upper chamber of the Transwell insert (8.0 µm pore size). The lower chamber was filled with medium containing 20% serum as a chemoattractant. After incubation at 37 °C for 24 h, cells that had not migrated through the pores were removed from the upper side of the Transwell membrane with a cotton swab. The cells on the lower side of the membrane were fixed with 4% paraformaldehyde, stained with 0.1% crystal violet, and then imaged using a light microscope. Migrated cells were quantified by counting the cells in five fields per membrane under 10 × magnification, and the mean number of cells was calculated.

### Detection of chemoresistance of cancer cells

Chemoresistance of cancer cells was assessed using MTT assay. Briefly, cells were seeded into 96-well plates and cultured overnight. The following day, cells were treated with cisplatin at different final concentrations (10, 25, 50, and 100 μg/mL). After 48 h of treatment, cell viability was assessed according to the protocol of the cell viability assay. The half maximal inhibitory concentration (IC_50_) values were calculated from the dose–response curves for each cell line.

### Silencing and rescue of gene expression

To knockdown *METTL3* or *METTL14* in cells, shRNAs (*METTL3*-shRNA, 5′-CGTCAGTATCTTGGGCAAGTT-3′; *METTL14*- shRNA, 5′-GCCGTGTTAAATAGCAAAGAT-3′) were cloned into pLVX-shRNA2 lentiviral vector. Then the recombinant vector was cotransfected with psPAX2 and pMD2.G into HEK293T cells using Lipofectamine 2000 (Invitrogen) to package the virus. At 48 h later, the viral suspension was collected for transfection into GCSCs. At 24 h after infection, GCSCs were screened by puromycin (0.5 μg/mL).

To rescue the expression of *METTL3* or *METTL14* in the *METTL3*-silenced or the *METTL14*-silenced stem cells, the *METTL3* gene or the *METTL14* gene was amplified using primers (*METTL3*, 5′-CCCAAGCTTTTCGAGAGGTGTCAGGGCTGGG-3′ and 5′-GCGG CCGCTAAACTATCAGAGCCATGGCTATGGATTCT; *METTL14*, 5′-CCCAAGC TTTCTGTTCGTAAGCTCCCGGTGA-3′ and 5′-GGATCCCGACAACTGCAAGC AAGGCTCGCT-3′) and inserted into pcDNA3.1 + vector. To avoid the targeting of recombinant *METTL3* or *METTL14* by *METTL3*-shRNA or *METTL14*-shRNA, the *METTL3* gene or the *METTL14* gene was mutated from C to T at position 1278 or from C to T at position 254. Then *METTL3*-knockdown or /*METTL14*-knockdown GCSCs were subjected to transfection with the recombinant plasmids.

To silence the expressions of *PSMA3-AS1*, *MIR22HG*, *AP1G1*, *SERTAD1*, *EEF1A1*, and *LRPPRC*, GCSCs were transfected with siRNAs (*PSMA3-AS1*-siRNA, 5′-GCAAAAATGCAAAATACCAGG-3′; *MIR22HG*-siRNA, 5′-ATATAAACATTACAGGCTGGG-3′; *AP1G1*-siRNA, 5′-TTGCGAGTCCTAGCCATAATT-3′; *SERTAD1*-siRNA, 5′-TATGTATGACAATGAA CTTTT-3′; *EEF1A1*-siRNA, 5′-CCAGAAGAGATATGAGGAATT-3′; *LRPPRC*- siRNA, 5′-CACCTTATGGACCGTGATTTT-3′). As a control, control siRNA (5′-T TCTCCGAACGTGTCACGTTT-3′) was also transfected into GCSCs.

### Quantitative real-time PCR

RNAs were extracted from either cells or the MeRIP products using Trizol extraction (TransGen). Subsequently, cDNA was synthesized using a reverse transcription system (Vazyme). qPCR was performed using SYBR Green PCR Master Mix (Vazyme) with primers (*PAPPA-AS1*, 5′-AGCCTCTTTTGCCTAATATCCTT-3′ and 5′-GCCACAGAAGAACCTTACCAG-3′; *PSMA3-AS1*, 5′-CTTGTCGGCGCCATTTTGTC-3′ and 5′-GGCCGCACAAAAACCAATCT-3′; *MIR22HG*, 5′-AGCGGACGCAGTGATTTGCT-3′ and 5′-TGGCAGCTTTAGCTGGGTCA-3′; *LINC00342*, 5′-ACTACAGTGGCAGACAGACC-3′ and 5′-CAGCCCAACTTTCTTTACTGTGTT-3′; *LINC01410*, 5′-CAAGAATGGCCCAAGCAGTC-3′ and 5′-CCCTCCTAGGTCCTGGTTGT-3′; *LINC00680*, 5′-AGTTGTTTGGGCTGTCGCT-3′ and 5′-GGGGGCAAGGCAAATCAATAC-3′; miR-101-3p, 5′-GCCGAGTACAGTACTGTGATA-3′ and 5′-CTCAACTGGTGTCGTGGA-3′; miR-4429, 5′-GCCGAGAAAAGCTGGGCT-3′ and 5′- CTCAACTGGTGTCGTGGA-3′; miR-411-3p, 5’-GCCGAGTATGTAACACGGTC-3′ and 5′- CTCAACTGGTGTCGTGGA-3′; miR-24-3p, 5′-GCCGAGTGGCTCAGTTCAGCA-3′ and 5′-CTCAACTGGTGTCGTGGA-3′; *TRPC4AP*, 5′-TGTCCATCCTGTTGAACCCG-3′ and 5′-CCTGTACCCAAAGACCTGGG-3′; *SERTAD1*, 5′-GGGTCATAGCTTGGGCTGTT-3′ and 5′- AGCAGCACACGGATTTGAGA-3′; *AP1G1*, 5′-TGCTTGCGCATTTCAGAAAGAA-3′ and 5′-CGTACCTGCAAAAAGGGGTC-3′; *EEF1A1*, 5′-TTTTCGCAACGGGTTTGCC-3′ and 5′-GATGGCCAGTAGTGGTGGAC-3′; *LRPPRC*, 5′-AGTGAAAGCATTCGCGGAGA-3′ and 5′-TTCATGGCCAATGCCTGGAT-3′; *GAPDH*, 5′-GGACCTGACCTGCCGTCTAG-3′ and 5′-GTAGCCCAGGATGCCCTTGA-3′; *U6*, 5′-CTCGCTTCGGCAGCACA-3′ and 5′-AACGCTTCACGAATTTGCGT). Expression data were consistently standardized to the internal control *GAPDH* or *U6* and the relative abundance of RNA were assessed by the △△Ct method.

### Examination of lncRNA stability

To assess the stability of lncRNAs, cells were exposed to actinomycin D (Sigma) at a final concentration of 80 nM. Cells were collected at different time points (0, 2, 4, 8, and 12 h) after treatment, followed by total RNA extraction and RT-qPCR.

### Site-specific methylation of lncRNAs in cells

Site-specific methylation of lncRNAs in cells was performed as described before [26]. Briefly the DNA fragment encoding the guide RNA targeting A1225 of *PSMA3-AS1* (5′-TACAGGTTATCTCAGAATATCTTTTTTGGC-3′) or A2041 of *MIR22HG* (5′-ATATTGGGTCTTATTTTTTTCTGTTATGGT-3′) was inserted into pC0043-PspCas13b crRNA backbone (Addgene). Subsequently the recombinant pC0043-PspCas13b crRNA backbone was cotransfected with the plasmid expressing METTL3 fused with catalytically inactivated Cas13 (dCas13b; Addgene) into cells (10^6^/well) using Lipofectamine 2000 (Invitrogen). The cells were cultured in Opti-MEM I Reduced Serum Media (Gibco). The cells were harvested 36 h post-transfection.

### Dot blot

Total RNAs extracted were heated at 98 °C for 3 min. After being chilled on ice immediately thereafter, RNAs were dropped onto a nitrocellulose membrane, followed by crosslink under ultraviolet. The membrane was incubated with blocking buffer (PBS, 0.02% Tween-20, 5% milk, pH7.4) for 1 h at room temperature. Successively, anti-m6A antibody was incubated with the membrane overnight at 4 °C. After washing with PBST, the membrane was incubated with the HRP-conjugated secondary antibody. The signals were examined using ECL kit (PerkinElmer).

### Dual-luciferase reporter assay

The recombinant pmirGLO vector inserted fragments of lncRNAs and synthesized miRNAs (GenePharma) were transfected together into GCSCs using Lipofectamine 2000. The scrambled sequence of a miRNA was used as a control. The luciferase activities were measured using the dual luciferase reporter assay kit (Vazyme) at 36 h post transfection.

### RNA pulldown assay

The biotin-labeled lncRNA probe (*PSMA3-AS1*, 5’- TACAAAATCCATCTGCT GACCCCTG-biotin-TEG-3′; *MIR22HG*, 5′-TCTCTACCACTGTCCCACACGCATC-biotin-TEG-3′; biotin-TEG, biotin with 15 atom triethylene glycol spacer) was synthesized by Hangzhou Youkang Biotechnology Inc. The lncRNA-biotin probe was incubated with streptavidin magnetic beads (Beyotime) in 1 × Binding & washing buffer (10 mM Tris–HCl, 1 mM EDTA, 2 M NaCl, 0.01–0.1% Tween-20, pH 7.5) for 30 min at room temperature. Then the lysate of cells were incubated with washed beads in for 2 h at 4 °C. Subsequently, miRNAs or proteins were extracted from the beads. The miRNA extraction was performed using mirVana miRNA Isolation Kit (Thermo Fisher Scientific) and analyzed by qPCR. The proteins were eluted using 0.1% SDS, followed by SDS-PAGE. Mass spectrometry was used for identification of binding proteins.

### Cell viability assay

At various time points posttreatment, cells were collected by centrifuging. Next, collected cells were exposed to CellTiter 96^®^ AQueous reagent (Promega, USA) and incubated at 37 °C for 2 h. The iMark Microplate Absorbance Reader (Bio-Rad) was used for detection of the absorbance of cells.

### Cell cycle analysis

Cells were incubated with prechilled 70% ethanol at 4 °C for 30 min, and then incubated with RNase A (Beyotime) at 37 °C for 20 min. Finally, incubation with PI (Yeasen) at 37 °C for 10 min was used for cell staining. The fluorescence intensity was measured using flow cytometry.

### Caspase 3/7 activity assays

1 × 10^6^ cells were exposed to 100 μL Caspase-Glo^®^ 3/7 reagent according to the instruction of the Caspase-Glo^®^ 3/7 Assay (Promega). After incubating at room temperature for 2 h, Glomax system (Promega) was used to examining the luminescence of cells.

### Tumorigenicity assay in vivo

GCSCs with the site-specific methylation of *PSMA3-AS1* or *MIR22HG* were constructed and then injected into mice. The guide RNA targeting *PSMA3-AS1* (5′-G TACAGGTTATCTCAGAATATCTTTTTTGGC-3′) or *MIR22HG* (5′-GATATTGG GTCTTATTTTTTTCTGTTATGGT-3′) was cloned into the pLVX-IRES-Neo lentiviral vector (Addgene) behind the cPPT site. *METTL3* was also cloned into the lentiviral vector as a fusion protein of Cas13b. The recombinant lentiviral vector was transfected into HEK293T cells to package the virus. At 48 h later, the viral suspension was collected for transfection into *METTL3*-silenced GCSCs and cultured for 24 h. The cells were then screened using G418 sulfate (Thermofisher) for 24 h. The living cells were collected and cultured for 48 h. Subsequently, 1 × 10^6^ cells were mixed with matrigel and subcutaneously injected into five female BALB/c mice at a volume of 100  μL per mouse. After 4 weeks, the mice were sacrificed and the solid tumors were dissected for subsequent use.

### Statistical analysis

All experiments were replicated a biological minimum of three times. One-way analysis of variation (ANOVA) and Student’s *t*-test were used to analyze the numerical data.

## Results

### N6-methyladenosine lncRNAs in GCSCs

**Fig. 1:**
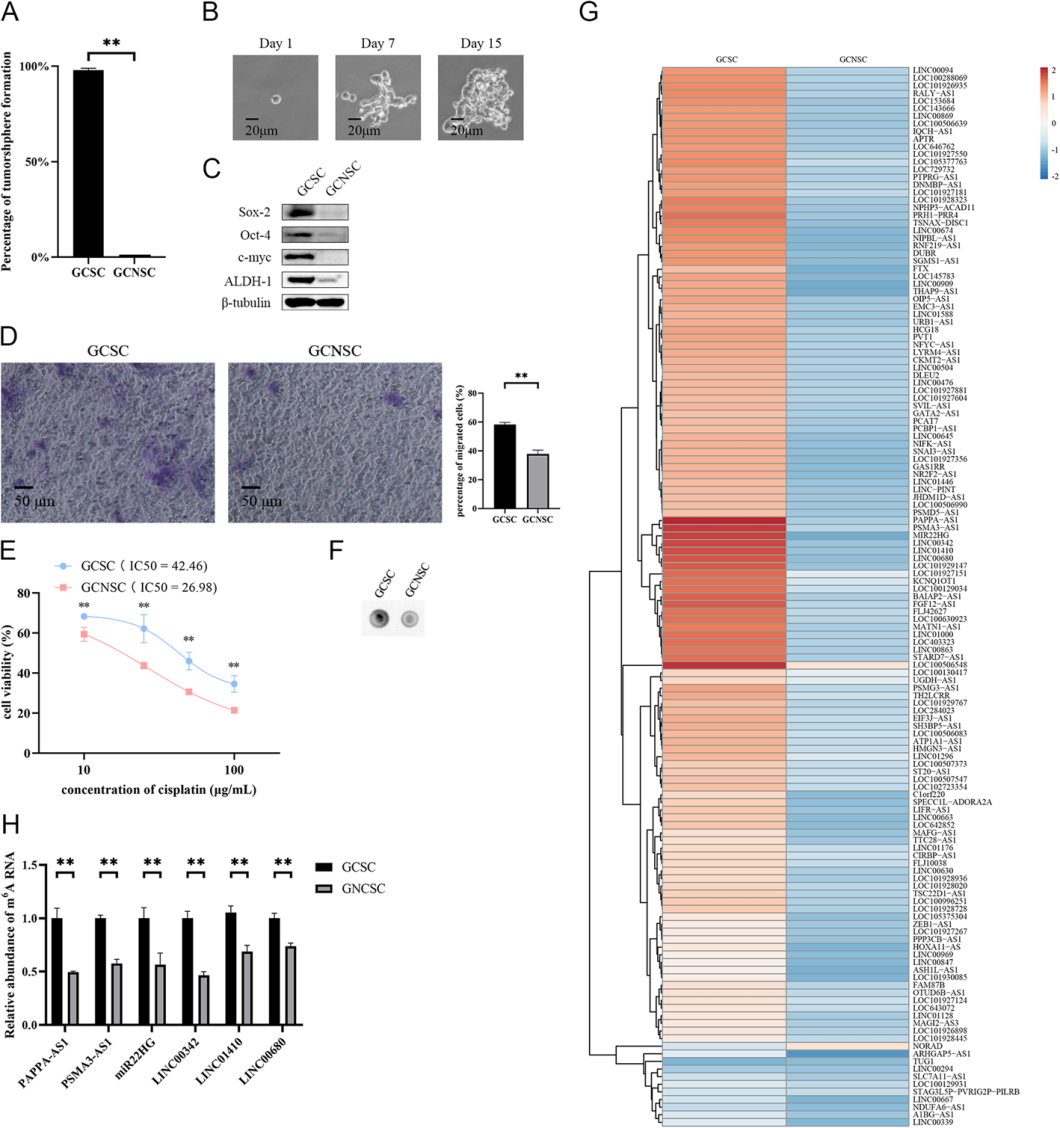
N6-methyladenosine lncRNAs in GCSCs. **A** Percentage of tumorsphere formation of GCSCs and GCNSCs. **B** Tumorsphere formation assay of a single cell from GCSCs; **C** detection of the expressions of stemness genes in GCSCs and GCNSCs with Western blot. **D** Efficiency of the migration of GCSCs. GCSCs and GCNSCs were subjected to Transwell migration assays to examine the cell migration. The representative images of the migrated cells are shown on the left. The percentage of the migrated cells is indicated on the right (**, *p* < 0.01). Scale bar, 50 μm. (**E**) Chemoresistance capacity of GCSCs. GCSCs or GCNSCs were treated with different concentrations of cisplatin. At 48 h after treatment, the cells were subjected to cell viability assays (**, *p* < 0.01). The half maximal inhibitory concentration (IC_50_) values were calculated. **F** The m6A modification of RNAs in GCSCs and GNCSCs. **G** Heatmap of the differentially expressed m6A-modified lncRNAs in GCSCs and GNCSCs. **H** Expression profiles of methylated lncRNAs in GCNSCs and GCSCs. **, *p* < 0.01

To reveal the relationship between the stemness of GCSCs and m6A modification of lncRNAs, the m6A-modified lncRNAs of GCSCs sorted from the solid tumors of a GC patient were characterized. The results indicated a tumorsphere formation rate of 97.9% for the identified potential GCSCs, whereas no tumorspheres were observed in the case of the GCNSCs (Fig. 1A). Correspondingly, a single cell from a tumorsphere of potential GCSCs had the capability to form another tumorsphere (Fig. 1B). Western blot analysis demonstrated a notable upregulation of some stemness genes in GCSCs (Fig. 1C). The migration efficiency and the chemoresistance capacity of GCSCs were significantly increased compared with those of GCNSCs (Fig. 1D and E). These data confirmed a successful isolation of GCSCs from solid tumors.

The results of dot blot using m6A-specific antibody revealed a considerably elevated m6A level in RNAs within GCSCs compared with GCNSCs (Fig. 1F), suggesting that the m6A modification of RNAs play a crucial role in GCSCs. Therefore, the m6A-modified lncRNAs of GCSCs were sequenced. On the basis of MeRIP-seq, the m6A level of 128 lncRNAs were elevated in GCSCs, yet the m6A level of 1 lncRNA was decreased in GCSCs (Fig. 1G). The MeRIP-seq data showed that the m6A levels of six lncRNAs (*PAPPA-AS1*, *PSMA3-AS1*, *MIR22HG*, *LINC00342*, *LINC01410*, and *LINC00680*) were significantly upregulated in GCSCs compared with those in GCNSCs (Fig. 1G). To confirm the expression profiles of the methylated lncRNAs in GCSCs, the m6A levels of these six lncRNAs were analyzed by MeRIP-qPCR. The results indicated that the six lncRNAs were significantly upregulated in GCSCs compared with the control (Fig. 1H), confirming the MeRIP-seq data.

Collectively, the findings unveiled a substantial increase in m6A levels of lncRNAs within GCSCs compared with those in GCNSCs, implying the essential roles of m6A modification of lncRNAs in GCSCs.

### Mechanism of lncRNA methylation in GCSCs

**Fig. 2:**
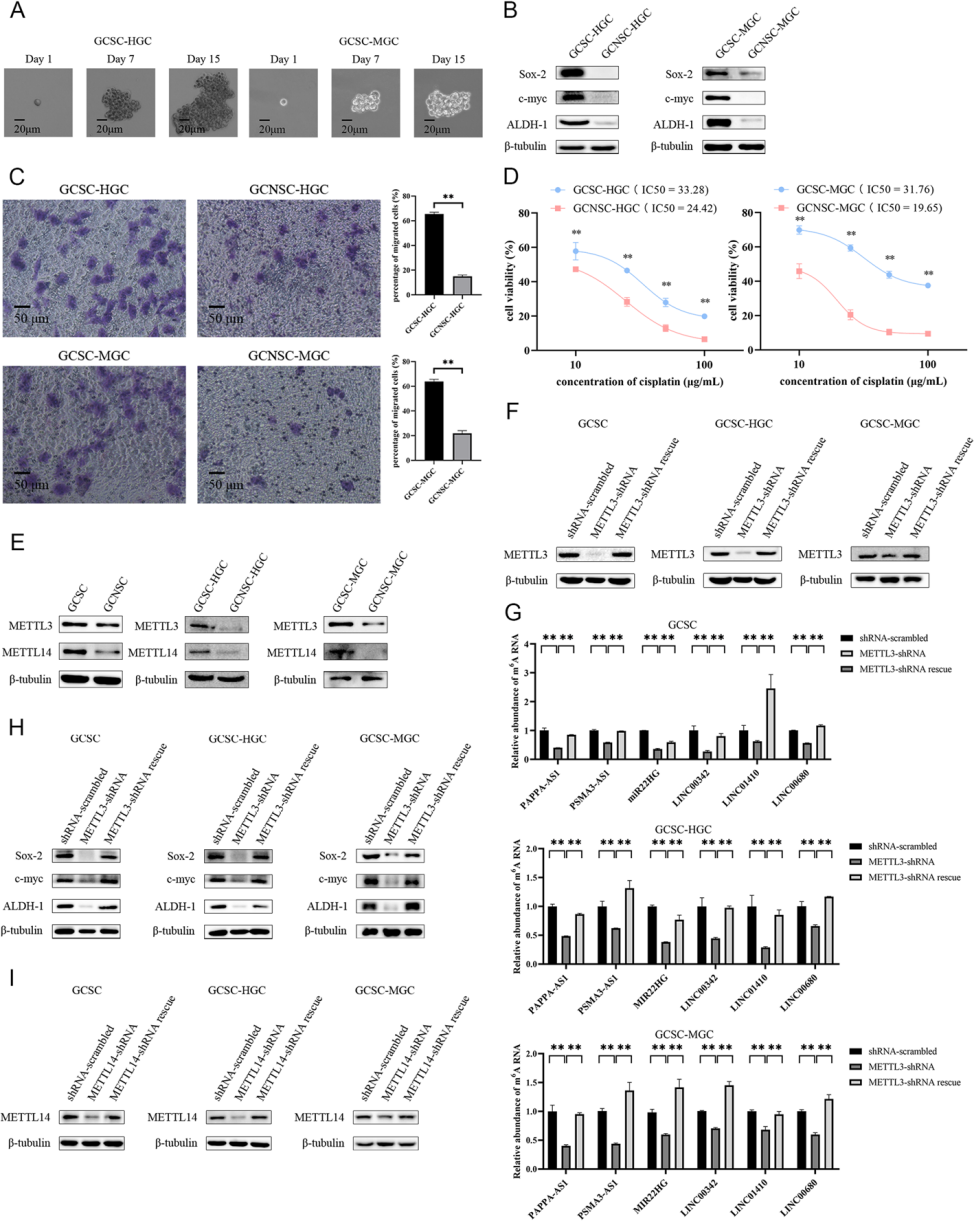

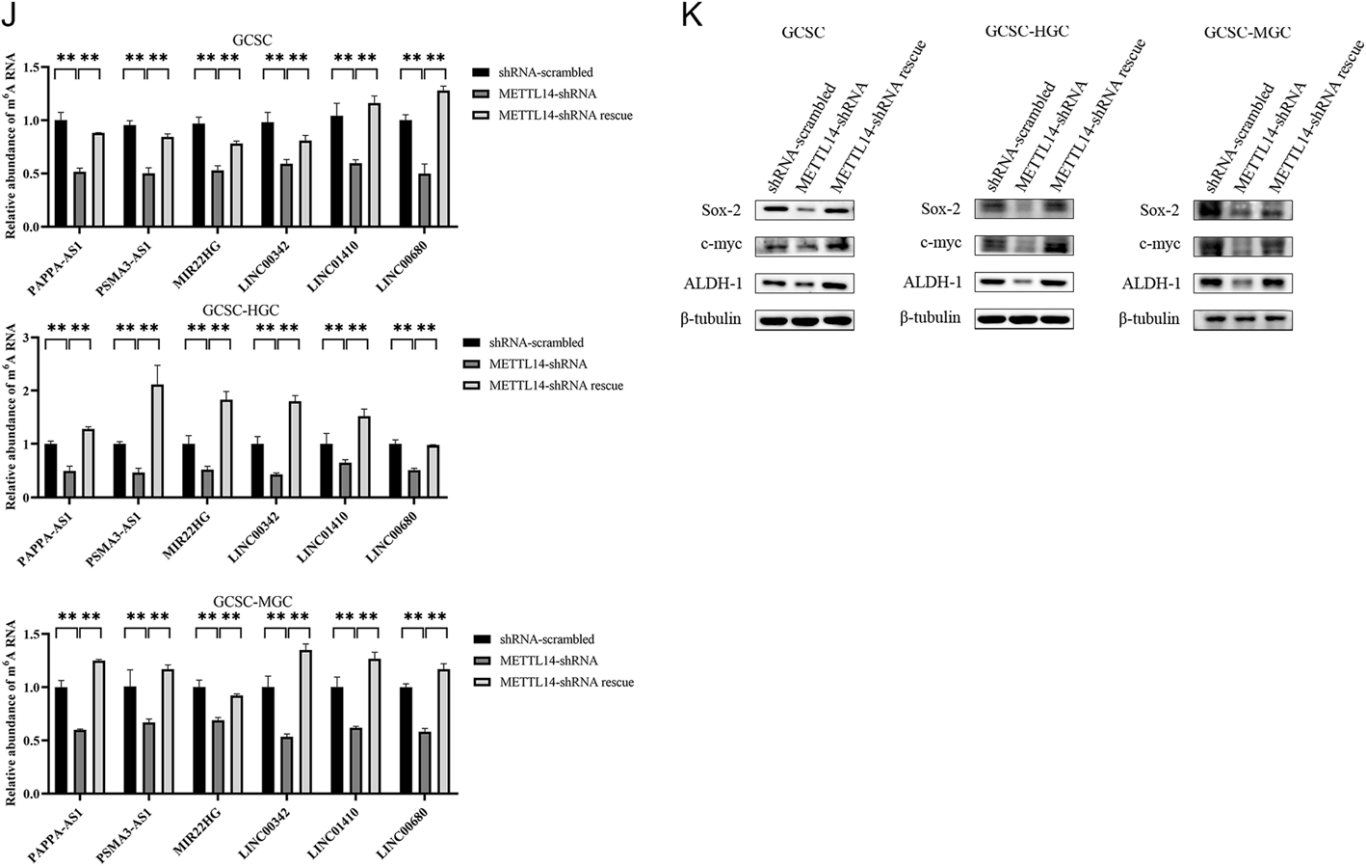
Mechanism of lncRNA methylation in GCSCs. **A** Tumorsphere formation assay of a single cell from the GCSCs sorted from HGC-27 (GCSC-HGC) and MGC-803 (GCSC-MGC). **B** Western blot analysis of the stemness genes in GCSC-HGC and GCSC-MGC. **C** Migration capacity of GCSCs (GCSC-HGC and GCSC-MGC). GCSC-HGC, GCNSC-HGC, GCSC-MGC, and GCNSC-MGC were analyzed with Transwell migration assays to examine the cell migration. The representative images of the migrated cells are indicated on the left. The percentage of the migrated cells is shown on the right (**, *p* < 0.01). Scale bar, 50 μm. **D** Efficiency of chemoresistance of GCSCs (GCSC-HGC and GCSC-MGC). GCSCs or GCNSCs were treated with cisplatin at different concentrations. At 48 h after treatment, the cells were characterized using cell viability assays (**, *p* < 0.01). The half maximal inhibitory concentration (IC_50_) values were calculated. **E** Examination of the expressions of methyltransferases METTL3 and METTL14 in GCSCs. **F** Western blot for METTL3 in GCSCs with *METTL3*-shRNA or *METTL3*-shRNA rescue. **G** m6A level of lncRNAs in GCSC with *METTL3*-shRNA or *METTL3*-shRNA rescue. **H** Detection of the expressions of stemness genes in the *METTL3*-silenced or rescued GCSCs using Western blot. β-tubulin was used as a control. **I** Western blot for *METTL14* in GCSCs with *METTL14*-shRNA or *METTL14*-shRNA rescue. **J** m6A level of lncRNAs in GCSC with *METTL14*-shRNA or *METTL14*-shRNA rescue. **K** Detection of the expressions of stemness genes in *METTL14*-silenced or rescued GCSCs by Western blot. **, *p* < 0.01

To elucidate the function of methylated lncRNAs in GCSCs, the GCSCs were further sorted from HGC-27 and MGC-803. Using flow cytometry, we sorted ALDH1-positive cells from HGC-27 and MGC-803, and the tumorsphere formation assay demonstrated that a tumorsphere could be formed by a single cell derived from the sorted ALDH1-positive cells of HGC-27 or MGC-803 (Fig. 2A). Meanwhile, Western blot analysis showed that the genes related to stemness were upgraded in the ALDH1-positive cells (Fig. 2B). The capacities of migration and chemoresistance of the ALDH1-positive cells were significantly increased compared with those of the control (Fig. 2C, D). These results showed a successful isolation of GCSCs from HGC-27 and MGC-803.

To reveal the mechanism of lncRNA methylation in GCSCs, the enzymes responsible for lncRNA methylation, including METTL3 and METTL14, the key methyltransferases for the m6A modification of mRNAs and lncRNAs, were characterized in GCSCs, GCSC-HGC, and GCSC-MGC. Western blot analysis revealed a substantial upregulation of METTL3 and METTL14 in three distinct GCSC types (Fig. 2E), suggesting the significant roles of *METTL3* and *METTL14* in the GCSCs.

To investigate the impact of METTL3/METTL14 on lncRNA methylation in GCSCs, sequence-specific shRNA (*METTL3*/*METTL14*-shRNA) was employed to knock down *METTL3*/*METTL14* in three GCSC types. Additionally, the silenced *METTL3*/*METTL14* was rescued (*METTL3*/*METTL14*-shRNA rescue) in the *METTL3*/*METTL14*-silenced GCSCs, and subsequent examination focused on lncRNA methylation. The results of western blot demonstrated the successful silencing or rescue of *METTL3*/*METTL14* in all three GCSC types (Fig. 2F, I). The silence of *METTL3*/*METTL14* caused a reduction of m6A level of lncRNAs, including *MIR22HG*, *LINC00342*, *PSMA3-AS1*, *LINC01410*, *PAPPA-AS1*, and *LINC00680*, in GCSCs, while *METTL3*/*METTL14*-shRNA rescue repaired the discrepancy of m6A level in lncRNAs (Fig. 2G, J).

To evaluate the impact of *METTL3*/*METTL14* on GCSCs, the protein levels of stemness genes were assessed in GCSCs with *METTL3*/*METTL14*-shRNA or *METTL3*/*METTL14*-shRNA rescue by western blot analysis. The findings demonstrated a significant reduction in protein levels of those genes in GCSCs with *METTL3*/*METTL14*-shRNA, whereas the expression levels of those genes in the *METTL3*/*METTL14*-shRNA rescued GCSCs were similar to those of the controls (Fig. 2H, K). These findings suggested that *METTL3*/*METTL14* enhanced the stemness of GCSCs.

In summary, *METTL3* and *METTL14* have demonstrated their crucial roles in lncRNA methylation of GCSCs, which could promote the stemness of GCSCs.

### Roles of methylated lncRNAs in GCSCs

**Fig. 3:**
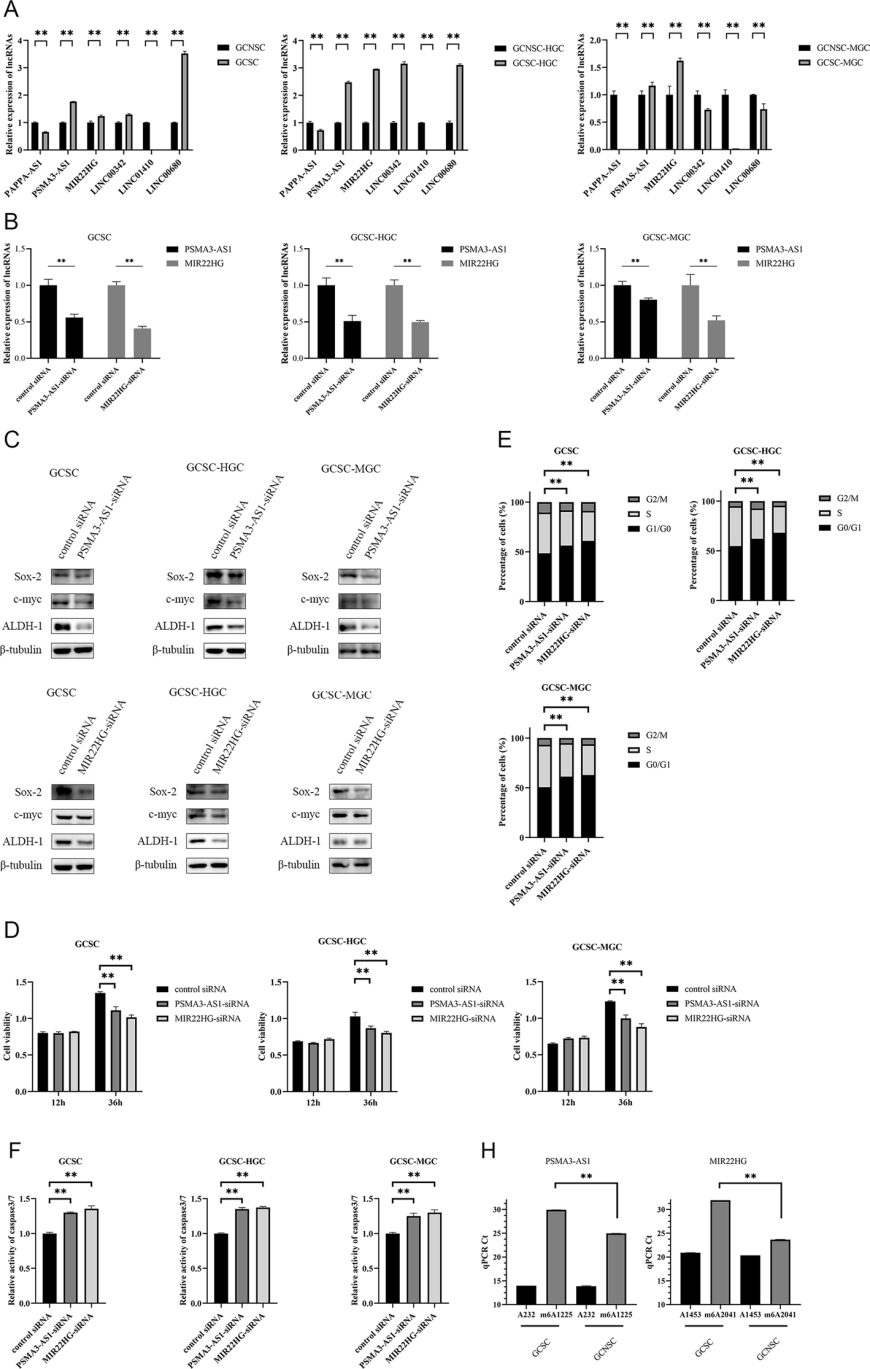

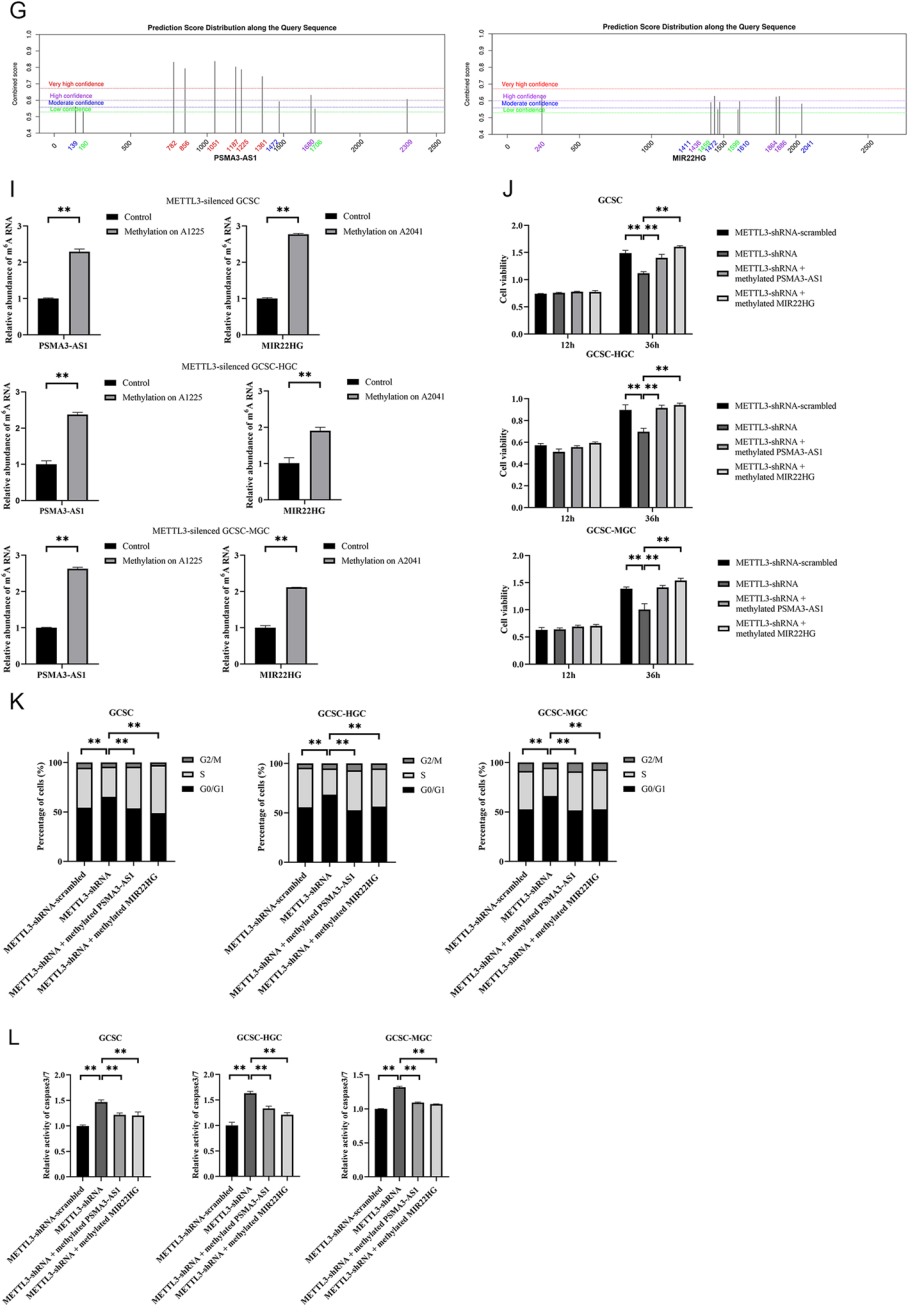

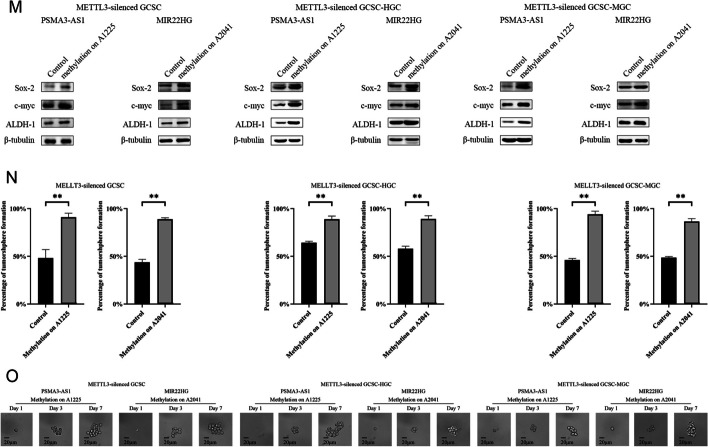
Roles of methylated lncRNAs in GCSCs. **A** Expression profiles of six lncRNAs (*PAPPA-AS1*, *PSMA3-AS1*, *MIR22HG*, *LINC00342*, *LINC01410*, and *LINC00680*) in GCSCs. **B** Validation of silencing of lncRNAs in GCSCs. **C** Detection of the expressions of stemness genes in GCSCs with lncRNAs-siRNA. **D** Cell viability for *PSMA3-AS1* or *MIR22HG* silencing GCSCs. **E** Cell cycle analysis of *PSMA3-AS1* or *MIR22HG*-silenced GCSCs. **F** Caspase-3/7 assay for *PSMA3-AS1* or *MIR22HG* silencing GCSCs. **G** Prediction of the methylation sites of lncRNAs. **H** The m6A levels of lncRNAs between GCSCs and GCNSCs. **I** The m6A levels of *METTL3*-silenced GCSCs with site-specific methylation of lncRNAs. **J** Cell viability for *METTL3*-silenced GCSCs with site-specific methylation of lncRNAs. **K** Cell cycle analysis of *METTL3*-silenced GCSCs with site-specific methylation of lncRNAs. **L** Caspase-3/7 assay for *METTL3*-silenced GCSCs with site-specific methylation of lncRNAs. **M** Western blot for stemness genes of *METTL3*-silenced GCSCs with site-specific methylation of lncRNAs. **N** The percentage of tumorsphere formation of *METTL3*-silenced GCSCs with site-specific methylation of lncRNAs. **O** Tumorsphere formation assay of a single cell from *METTL3*-silenced GCSCs with methylated lncRNAs. **, *p* < 0.01

On the basis of the MeRIP-Seq data, six lncRNAs (*PAPPA-AS1*, *PSMA3-AS1*, *MIR22HG*, *LINC00342*, *LINC01410*, and *LINC00680*) had a high m6A level in GCSCs. To further elucidate the functions of methylated lncRNAs in GCSCs, the expression profiles of six lncRNAs were investigated. The results showed that two lncRNAs (*PSMA3-AS1* and *MIR22HG*) were substantially elevated in all GCSCs compared with the corresponding GCNSCs (Fig. 3A), suggesting the crucial roles played by these lncRNAs in GCSCs.

To evaluate the impact of lncRNAs on GCSCs, *PSMA3-AS1* or *MIR22HG* was knocked down in GCSCs, followed by examining the stemness of knockdown GCSCs. The RT-qPCR data demonstrated that *PSMA3-AS1* and *MIR22HG* were respectively silenced in three types of GCSCs (Fig. 3B). The silencing of *PSMA3-AS1* or *MIR22HG* caused a significant downregulation of the stemness markers in GCSCs (Fig. 3C), showing the important roles of *PSMA3-AS1* or *MIR22HG* in GCSCs. And the results of the cell viability assay showed that the *PSMA3-AS1* or *MIR22HG* downregulation significantly reduced the viability of GCSCs (Fig. 3D). The suppression of GCSCs’ viability mediated by the *PSMA3-AS1* or *MIR22HG* silencing resulted from cell cycle arrest in the G0/G1 phase, subsequently leading to apoptosis of GCSCs (Fig. 3E, F). These findings indicated that *PSMA3-AS1* and *MIR22HG* exert positive influences on GCSCs.

To investigate the impact of m6A modification on lncRNAs in GCSCs, we initially predicted the m6A sites within the lncRNAs *PSMA3-AS1* and *MIR22HG* by SRAMP (http://www.cuilab.cn/sramp). The analysis revealed the presence of 12 potential m6A sites in *PSMA3-AS1* and 10 in *MIR22HG* (Fig. 3G). According a previous report, single-base elongation- and ligation-based qPCR amplification analysis (SELECT) was performed to determine the m6A sites of lncRNAs [27].The data of qPCR analysis confirmed that 11 and 8 of the predicted m6A sites of *PSMA3-AS1* and *MIR22HG* were methylated in GCSCs, respectively (Additional file 1: Fig. S1A). Among the m6A sites of lncRNAs, the N6-methyladenosine modification level at position 1225 of *PSMA3-AS1* (m6A1225) or at position 2041 of *MIR22HG* (m6A2041) was significantly increased in GCSCs compared with GCNSCs (Fig. 3H), implying that the site-specific methylated adenosine of *PSMA3-AS1* and *MIR22HG* play a significant role in GCSCs.

To elucidate the impact of the site-specific m6A modification of lncRNAs on GCSCs, the A1225 site of *PSMA3-AS1* and the A2041 site of *MIR22HG* were specifically methylated in the *METTL3*-silenced GCSCs and then the methylation on those lncRNAs were confirmed (Fig. 3I). Meanwhile, the results of the cell viability assay indicated that silencing of *METTL3* obviously impaired the viability of GCSCs, while the methylation of A1225 of *PSMA3-AS1* (*METTL3*-shRNA + methylated *PSMA3-AS1*) or A2041 of *MIR22HG* (*METTL3*-shRNA + methylated *MIR22HG*) significantly increased the viability of *METTL3*-silenced GCSCs compared with *METTL3*-silenced GCSCs (*METTL3*-shRNA) (Fig. 3J), implying that the site-specific m6A modification of lncRNAs could promote the proliferation of GCSCs. The data of cell cycle analysis revealed that GCSCs with *METTL3*-shRNA induced cell cycle arrest in G1/G0 phase, and the methylation of A1225 of *PSMA3-AS1* or A2041 of *MIR22HG* recovered the cell cycle of GCSCs (Fig. 3K). Correspondingly, the *METTL3* knockdown-mediated cell cycle arrest activated apoptosis of GCSCs, whereas the apoptotic activity of *METTL3*-silenced GCSCs with the methylation of A1225 of *PSMA3-AS1* (*METTL3*-shRNA + methylated *PSMA3-AS1*) or A2041 of *MIR22HG* (*METTL3*-shRNA + methylated *MIR22HG*) was commensurate with that of the control (*METTL3*-shRNA-scrambled; Fig. 3L). Evidently, the site-specific m6A modification of lncRNAs was required for the proliferation of GCSCs.

Furthermore, the western blots analysis revealed a significant elevation of stemness in *METTL3*-silenced GCSCs with methylation of the A1225 site of *PSMA3-AS1* or the A2041 site of *MIR22HG* (Fig 3M). Consistently, the methylation of *PSMA3-AS1* or *MIR22HG* significantly promoted the tumorsphere formation of the *METTL3*-silenced GCSCs (Fig 3N and O). These data unveiled a positive contribution of lncRNA methylation to stemness of GCSCs.

Together, these data demonstrated that site-specific m6A modification of lncRNAs (*PSMA3-AS1* and *MIR22HG*) was required for the proliferation of GCSCs via suppressing apoptosis and promoting the stemness of GCSCs.

### Influence of m6A modification on the stability of lncRNAs

**Fig. 4:**
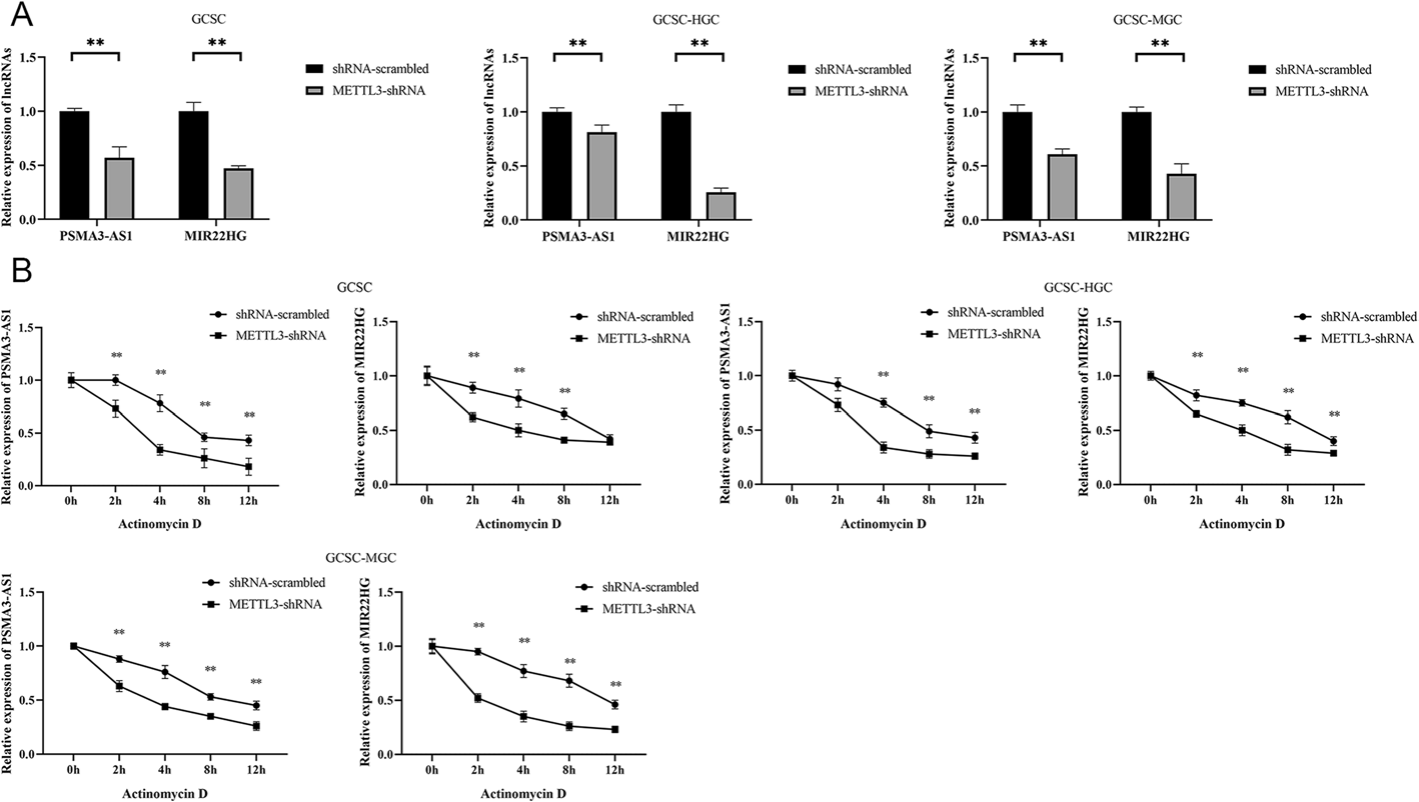
Influence of m6A modification on the stability of lncRNAs. **A** Relative expression level of lncRNAs *PSMA3-AS1* and *MIR22HG* in *METTL3*-silenced GCSCs. **B** Examination of lncRNAs stability in *METTL3*-silenced GCSCs by treating with actinomycin D. **, *p* < 0.01

To investigate the effect of m6A modification on lncRNA stability, the RNA levels of *PSMA3-AS1* and *MIR22HG* in *METTL3*-silenced GCSCs were determined. A considerable decrease of the RNA levels of *PSMA3-AS1* and *MIR22HG* were exhibited in *METTL3*-silenced GCSCs (Fig. 4A), intimating that m6A modification could enhance the stability of lncRNAs.

To further validate the influence of m6A modification on lncRNAs stability, the *METTL3*-silenced GCSCs were exposed to actinomycin D, followed by the assessment of lncRNAs enrichment. The findings indicated elevated expression levels of *PSMA3-AS1* and *MIR22HG* in *METTL3*-silenced GCSCs treated with actinomycin D than those in GCSCs (Fig. 4B). It can be inferred that m6A modification could stabilize *PSMA3-AS1* and *MIR22HG* in GCSCs.

### Underlying mechanism of lncRNAs in GCSCs

**Fig. 5:**
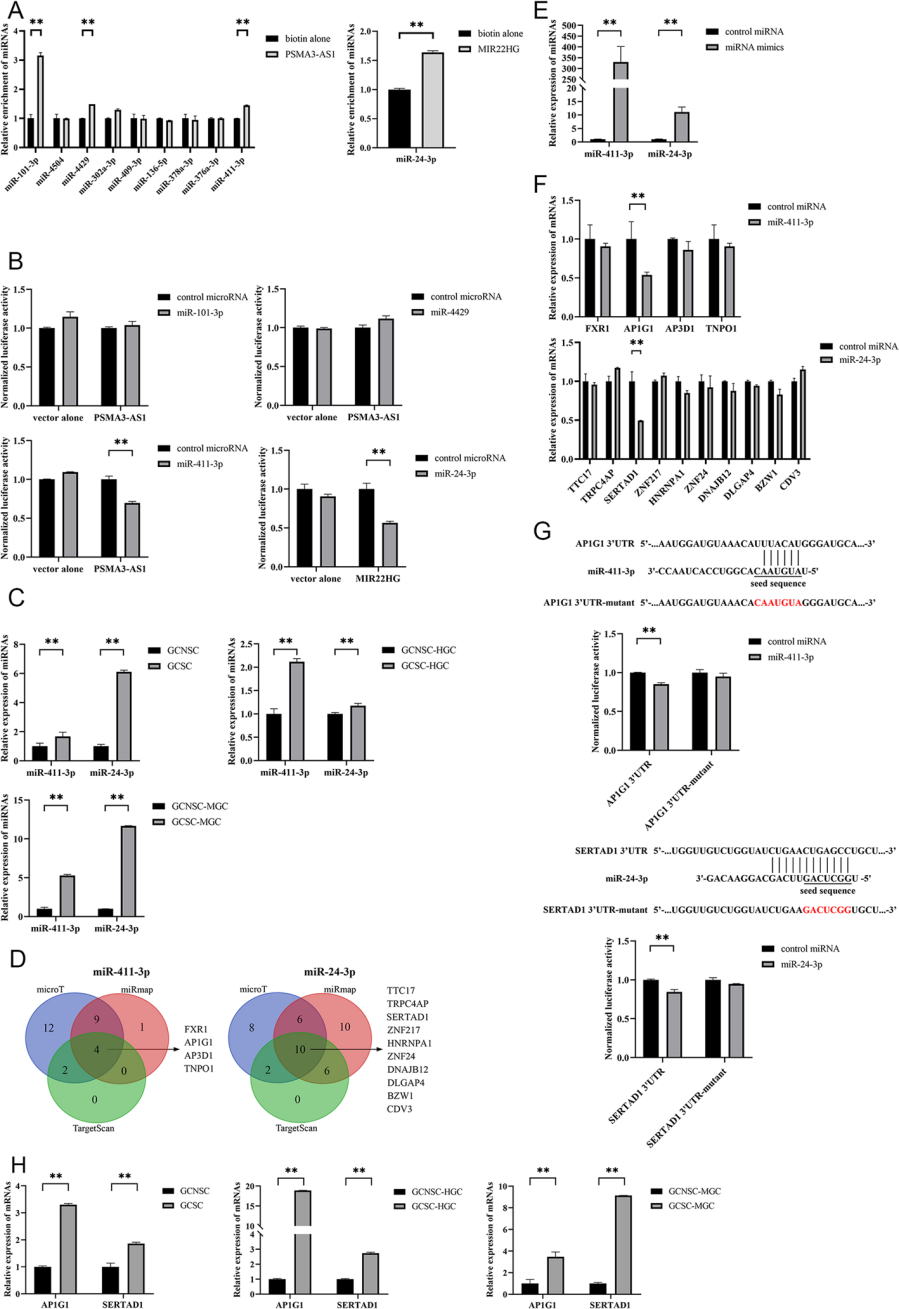

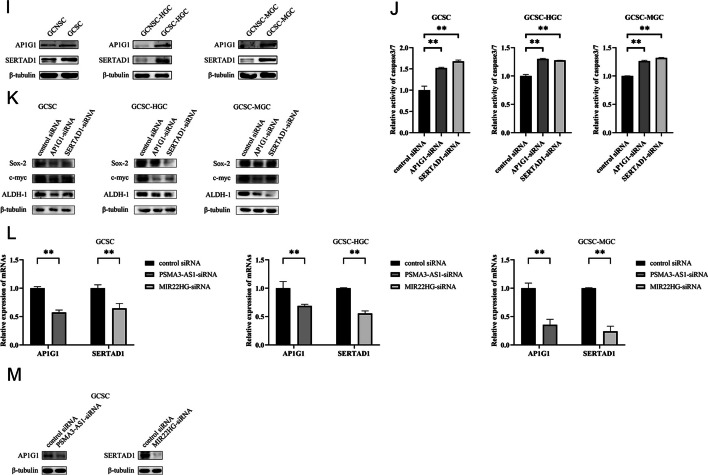
Underlying mechanism of lncRNAs in GCSCs. **A** The relative enrichment of miRNAs interacted with lncRNAs in GCSCs. **B** Dual-luciferase reporter assay for potential miRNAs-lncRNAs interaction in GCSCs. **C** The expression profiles of miR-411-3p and miR-24-3p in GCSCs and GCNSCs. **D** Prediction of potential target genes of miRNAs using microT, miRmap, and TargetScan. **E** Validation for overexpression of miRNAs in GCSCs. **F** Detection of the expression levels of target genes in the miRNA-overexpressed GCSCs. **G** Validation of interaction between miRNAs and target genes by dual-luciferase reporter assay. **H** The expression levels of target genes of miRNAs in GCSCs and GCNSCs. **I** Western blot for target genes of miRNAs in GCSCs and GCNSCs. **J** Caspase-3/7 assay for *AP1G1* or *SERTAD1* silencing GCSCs. **K** Western blot for stemness genes in *AP1G1* or *SERTAD1* silencing GCSCs. **L** The expression level of AP1G1 or SERTAD1 in *PSMA3-AS1* or *MIR22HG* silencing GCSCs. **M** Western blot analysis for target genes of miRNAs in *PSMA3-AS1* or *MIR22HG*-silenced GCSCs. **, *p* < 0.01

Generally, lncRNAs function by binding to miRNAs. On the basis of literature and prediction using ENCORI and DIANA-LncBase, the potential miRNAs targeted by *PSMA3-AS1* or *MIR22HG* were obtained (Additional file 1: Table S1). The results of lncRNA pulldown assays showed that miR-101, miR-4429, and miR-411-3p were notably enriched in the pulled down RNAs using *PSMA3-AS1*, while miR-24-3p was significantly enriched in the pulled down RNAs using *MIR22HG* (Fig. 5A). Thereafter, the dual luciferase reporter assays revealed that miR-411-3p or miR-24-3p significantly reduced the luciferase activity of GCSC cotransfected with miR-411-3p and pmirGLO-*PSMA3-AS1* or miR-24-3p and pmirGLO-*MIR22HG* (Fig. 5B), suggesting the direct interaction between miR-411-3p and *PSMA3-AS1*, in a similar manner to miR-24-3p and *MIR22HG*.

To elucidate the roles of miR-411-3p and miR-24-3p in GCSCs, we examined the expression profiles of these miRNAs in both GCSCs and GCNSCs. The findings indicated an obvious upregulation of miR-411-3p and miR-24-3p in GCSCs (Fig. 5C), suggesting that these two miRNAs played important roles in GCSCs.

To further explore the roles of the miRNAs interacting with lncRNAs, the potential targets of miRNAs were predicted using microT, miRmap, and TargetScan algorithms. The results showed that four genes were the potential targets of miR-411-3p, while ten genes might be targeted by miR-24-3p (Fig. 5D). To confirm the target genes of miRNAs, the change of expression of target genes were examined after transfecting with miR-411-3p or miR-24-3p in GCSC. Upon overexpression of miR-411-3p or miR-24-3p in GCSC (Fig. 5E), the expression level of *AP1G1* or *TRPC4AP* and *SERTAD1* was substantially diminished (Fig. 5F), implying that miR-411-3p or miR-24-3p might target *AP1G1* or *TRPC4AP* and *SERTAD1*. And the dual luciferase assays were performed and the data demonstrated that *AP1G1* or *SERTAD1* was the gene targeted by miR-411-3p or miR-24-3p (Fig. 5G).

To explore whether the target genes of miRNAs had effects on GCSCs, the expression levels of *AP1G1* and *SERTAD1* in GCSCs and GCNSCs were assessed. The findings indicated a remarkable upregulation of AP1G1 and SERTAD1 in GCSCs (Fig. 5H and I), suggesting that *AP1G1* and *SERTAD1* might play essential roles in GCSCs. The *AP1G1* or *SERTAD1* silencing induced apoptosis of GCSC (Fig. 5J) and downregulated the protein abundance of stemness genes in GCSC (Fig. 5K), showing that *AP1G1* and *SERTAD1* were required for shaping GCSC characteristics. As reported, *AP1G1* and *SERTAD1* promote gastric cancer progression [28, 29]. In this context, *AP1G1* and *SERTAD1* could promote tumorigenesis of GCSCs.

The m6A sites of lncRNAs *PSMA3-AS1* and *MIR22HG* were far from the base sequences bound to the seed sequences of miRNAs. Thus, the m6A modification of lncRNAs did not affect the interaction between *PSMA3-AS1* and miR-411-3p, similarly to *MIR22HG* and miR-24-3p. To assess whether *PSMA3-AS1* or *MIR22HG* functioned as a ceRNA, the expression profiles of *AP1G1* or *SERTAD1* were determined in the *PSMA3-AS1* or *MIR22HG*-silenced GCSCs. The findings revealed that the *PSMA3-AS1* or *MIR22HG* silencing noticeably decreased the expression level of miRNA-targeted mRNA of *AP1G1* or *SERTAD1* (Fig. 5L and M), indicating that lncRNAs *PSMA3-AS1* and *MIR22HG* functioned as ceRNAs in GCSCs.

Collectively, these findings demonstrated that lncRNA *PSMA3-AS1* or *MIR22HG* promoted the stemness of GCSCs via the *PSMA3-AS1*–miR-411-3p–*AP1G1* or *MIR22HG*– and miR-24-3p–*SERTAD1* axis.

### Interactions between m6A-modified lncRNAs and proteins

**Fig. 6:**
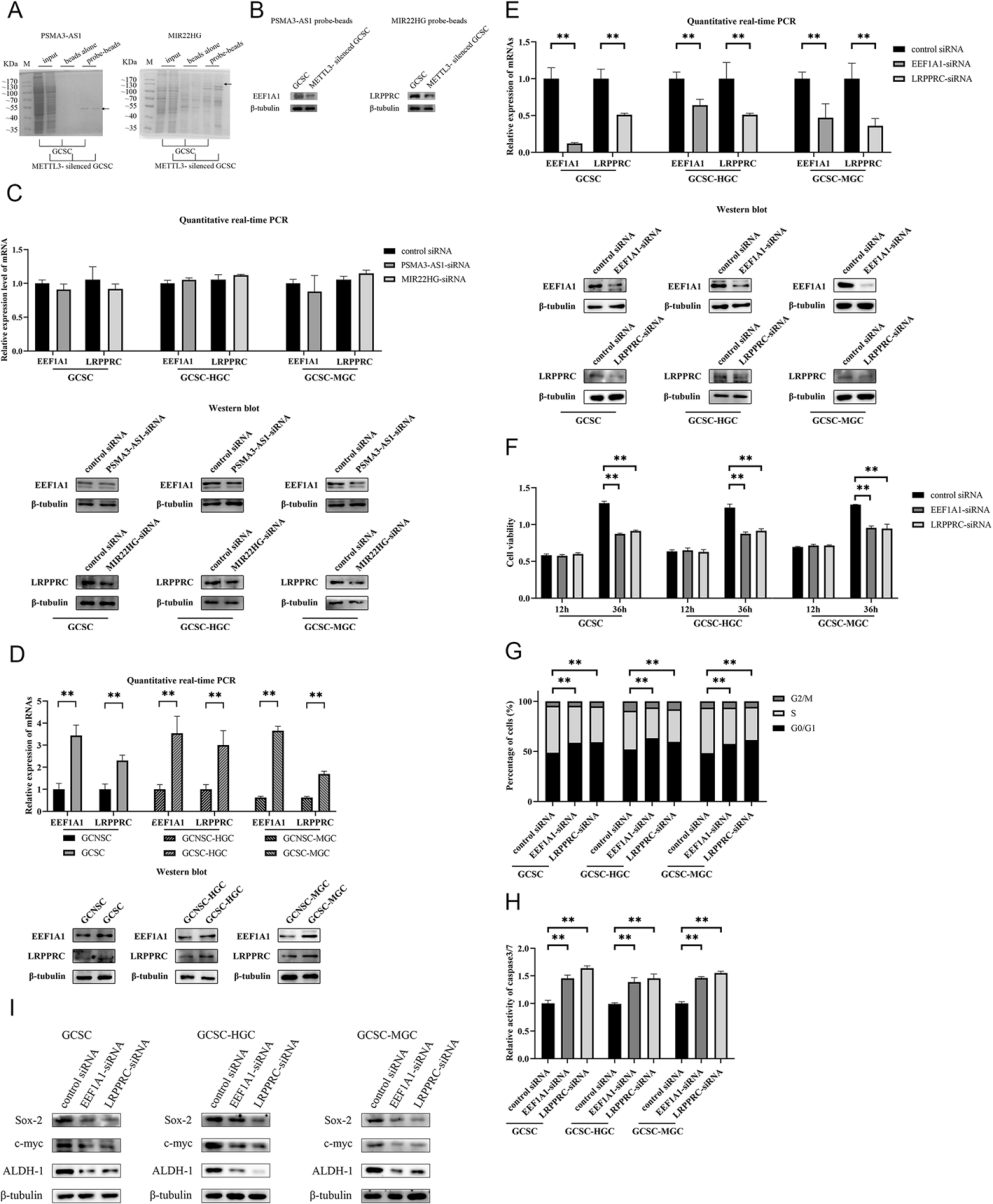
Interactions between m6A-modified lncRNAs and proteins. **A** SDS-PAGE for lncRNAs pulldown assay. The arrows indicate the proteins identified. M, protein marker. **B** Western blot analysis of the proteins bound to lncRNAs. **C** Detection of the stability of proteins bound to lncRNAs in *PSMA3-AS1* or *MIR22HG* silencing GCSCs. **D** The levels of mRNAs and proteins of *EEF1A1* and *LRPPRC* in GCSCs. **E** Validation of silencing of *EEF1A1* or *LRPPRC* in GCSCs by RT-qPCR and Western blot assay. **F** Cell viability for *EEF1A1* or *LRPPRC* silencing GCSCs. **G** Cell cycle analysis of *EEF1A1* or *LRPPRC* silencing GCSCs. **H** Caspase-3/7 assay of *EEF1A1* or *LRPPRC* silencing GCSCs. **I** Western blot for stemness genes of *EEF1A1* or *LRPPRC* silencing GCSCs. **, *p* < 0.01

To reveal the proteins that interacted with lncRNAs, lncRNA pulldown assays were performed. The results exhibited that a specific protein from GCSCs, identified to be EFF1A1 or LRPPRC, was bound to *PSMA3-AS1* or *MIR22HG* (Fig. 6A). However, the protein level from the *METTL3*-silenced GCSCs was lower than that from GCSCs (Fig. 1A), connoting that the methylation of *PSMA3-AS1* or *MIR22HG* affected the EFF1A1–*PSMA3-AS1* and LRPPRC–*MIR22HG* interactions. Western blots confirmed the interaction between EFF1A1 and *PSMA3-AS1* or LRPPRC and *MIR22HG* and the lncRNA–protein interaction was weakened by lncRNA demethylation (Fig. 6B). These data indicated that lncRNA methylation promoted the interaction between lncRNA and protein.

To unveil the impact of lncRNA methylation on the proteins bound to lncRNAs, protein stability was detected and the findings showed that *PSMA3-AS1* or *MIR22HG* silencing decreased the EFF1A1 or LRPPRC protein level in GCSCs compared with the controls (Fig. 6C). However, *PSMA3-AS1* or *MIR22HG* silencing had no impact on the *EFF1A1* or *LRPPRC* mRNA level (Fig. 6C). These data demonstrated that the methylation of lncRNAs enhanced the stability of the proteins bound to *PSMA3-AS1* or *MIR22HG*.

To investigate the roles of *EEF1A1* and *LRPPRC* in GCSCs, the differential expression of *EEF1A1* or *LRPPRC* in GCSCs and GCNSCs was evaluated. The results showed that at both RNA and protein levels, abundance of *EEF1A1* and *LRPPRC* were considerably elevated in GCSCs (Fig. 6D), suggesting that *EEF1A1* and *LRPPRC* played important roles in GCSCs. To reveal the function of *EEF1A1* and *LRPPRC* in GCSCs, *EEF1A1* and *LRPPRC* were knocked down in GCSCs (Fig. 6E). *EEF1A1* or *LRPPRC* silencing led to the inhibition of GCSCs’ viability (Fig. 6F) and caused cell cycle arrest in G0/G1 phase of GCSCs, which triggered apoptosis of GCSCs (Fig. 6G, H). Consistently, the expression of stemness genes were impaired in *EEF1A1*-silenced or *LRPPRC*-silenced GCSCs (Fig. 6I). These data revealed the influential role of EEF1A1 and LRPPRC in maintaining stemness and proliferation of GCSCs.

Taken together, these results implied that the methylated *PSMA3-AS1* or *MIR22HG* was interacted with EFF1A1 or LRPPRC protein to enhance the protein stability, leading to the suppression of apoptosis and the promotion of stemness of GCSCs.

### Influence of m6A modification of lncRNAs on tumorigenesis of GCSCs in vivo

**Fig. 7:**
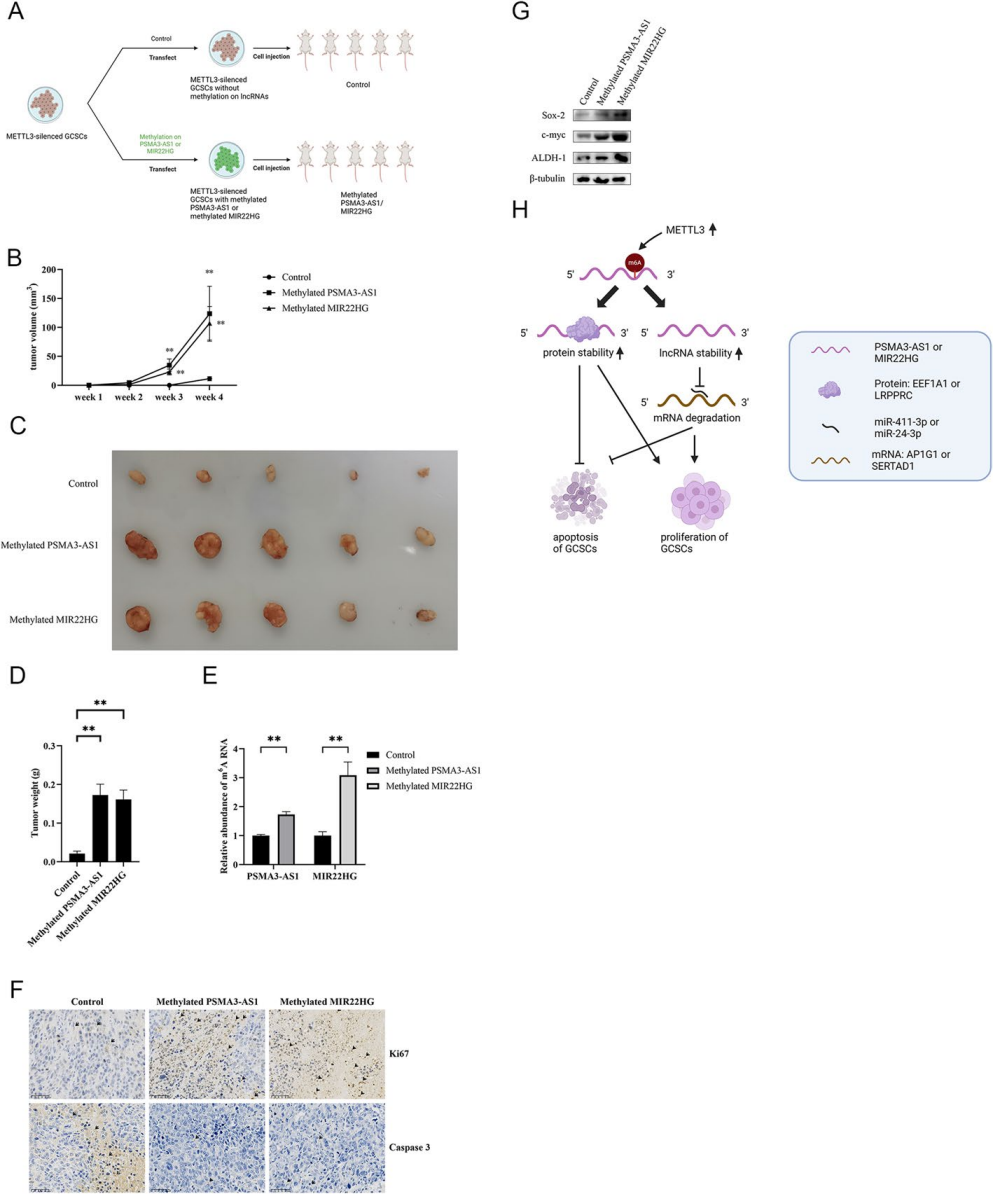
Influence of m6A modification of lncRNAs on tumorigenesis of GCSCs in vivo. **A** Schematic diagram for model mice with lncRNA-methylated GCSCs. **B** The tumor volume of the xenograft mice injected with *METTL3*-silenced GCSCs alone (control) or *METTL3*-silenced GCSCs with methylated *PSMA3-AS1* or *MIR22HG*. **C** The size of solid tumors of the mice with *METTL3*-silenced GCSCs alone (control) or *METTL3*-silenced GCSCs with methylated *PSMA3-AS1* or *MIR22HG*. **D** The weight of solid tumors of mice with *METTL3*-silenced GCSCs alone (control) or *METTL3*-silenced GCSCs with methylated *PSMA3-AS1* or *MIR22HG*. **E** m6A levels of lncRNAs in the solid tumors of the mice injected with *METTL3*-silenced GCSCs alone (control) or *METTL3*-silenced GCSCs with methylated *PSMA3-AS1* or *MIR22HG*. **F** Immunohistochemical analysis of the expressions of Ki67 and Caspase 3 in the solid tumors of the mice injected with *METTL3*-silenced GCSCs alone (control) or *METTL3*-silenced GCSCs with methylated *PSMA3-AS1* or *MIR22HG*. **G** Detection of the expressions of stemness genes in solid tumors of the mice treated with the methylated *PSMA3-AS1* or *MIR22HG* using Western blot. **H** Model for the roles of the methylated *PSMA3-AS1* and *MIR22HG* in GCSCs. **, *p* < 0.01

To investigate the influence of methylated *PSMA3-AS1* and *MIR22HG* on tumorigenesis of GCSCs in vivo, *METTL3*-silenced GCSC with or without the m6A modification of *PSMA3-AS1* or *MIR22HG* were injected into BALB/c mice (Fig. 7A). The tumor volume exhibited a more rapid growth in the mice injected with *METTL3*-silenced GCSCs containing the methylated *PSMA3-AS1* or *MIR22HG* than mice without the lncRNA methylation (Fig. 7B). Uniformly, much bigger and heavier tumors were developed in the mice with methylated *PSMA3-AS1* or *MIR22HG* (Fig. 7C and D). Thus, it can be inferred that m6A modification of *PSMA3-AS1* or *MIR22HG* prominently contributes to the promotion of tumorigenesis in GCSCs in vivo.

Then m6A level of lncRNAs were detected for confirming the methylation of lncRNAs in solid tumors (Fig. 7E). To assess the function of lncRNA methylation on solid tumors, the apoptosis and proliferation of solid tumors were examined using immunohistochemical analysis. The immunohistochemical analysis data exhibited that Caspase 3 was dramatically reduced in the solid tumors of mice with methylated *PSMA3-AS1* or *MIR22HG*, whereas Ki67 was upregulated (Fig. 7F), implying that the methylation of lncRNAs promoted tumor growth by suppressing apoptosis in vivo. Furthermore, the expression of stemness genes in the tumors of mice with the treatment of methylated *PSMA3-AS1* or *MIR22HG* were upregulated (Fig. 7G). These results illustrated the positive contribution of lncRNA methylation to tumor progression in vivo.

In conclusion, these results underscore the potential of m6A modification in lncRNAs (*PSMA3-AS1* and *MIR22HG*) to drive tumorigenesis in GCSCs through both miRNA-mediated and protein-mediated pathways (Fig. 7H).

## Discussion

The dysregulation of lncRNAs has been increasingly recognized as a significant contributor to tumorigenesis [30]. Although the precise mechanisms through which lncRNAs exert their functions are still being elucidated, it is established that lncRNAs play a role in modulating gene expression at diverse levels [31, 32]. Comprehending the complex interplay between lncRNAs and tumorigenesis is essential for unraveling the underlying mechanisms driving cancer progression [32]. Owing to the importance of lncRNAs, the epigenetic modifications of lncRNAs, including RNA methylation, have emerged as crucial mechanisms modulating lncRNAs’ roles in tumorigenesis, shedding light on the regulatory approaches that contribute to cancer pathogenesis [33, 34]. The methylation of lncRNAs, specifically m6A, is a prominent posttranscriptional modification of lncRNAs [33, 34]. Accumulating evidence highlights the profound influence of m6A modification of lncRNAs on the regulation of the stability, subcellular localization, and interactions with miRNAs and RNA-binding proteins [33, 34]. The m6A modification of lncRNAs can facilitate the recognition by specific reader proteins, leading to either enhanced or reduced stability, thereby shaping lncRNA abundance and availability [35]. The m6A modification landscape can impact the transport and spatial distribution of lncRNAs within distinct cellular compartments, orchestrating their interactions with target genes or proteins [35]. However, the involvement of m6A modification in lncRNA-mediated tumorigenesis remains largely unexplored. In this study, based on the global screening and characterization of the methylated lncRNAs in GCSCs, the site-specific m6A modification was found in two lncRNAs *PSMA3-AS1* and *MIR22HG* of GCSCs. The methylated *PSMA3-AS1* and *MIR22HG*, which were upregulated in GCSCs sorted from the solid tumors of a patient with gastric cancer and two distinct gastric cancer cell lines (HGC-27 and MGC-803), suppressed apoptosis and promoted the stemness of three types of GCSCs, thus promoting tumorigenesis. In this context, our findings revealed the requirement of the site-specific methylation of lncRNAs in tumorigenesis, shedding light on potential therapeutic targets for gastric cancer treatment.

Numerous studies have provided compelling evidence to demonstrate the popular roles of lncRNAs as ceRNAs in cancer development [36]. In this study, the results indicated that the site-specific m6A modification of *PSMA3-AS1* and *MIR22HG* enhanced the stability of lncRNAs in GCSCs, thus having a promotive role on *PSMA3-AS1* and *MIR22HG* as ceRNAs in tumorigenesis of gastric cancer. Accordingly, our discoveries unveiled an innovative mechanism of lncRNA-mediated ceRNAs. In our study, it was found that the methylated but not the unmethylated *PSMA3-AS1* or *MIR22HG* could interact with EEF1A1 or LRPPRC protein in GCSCs. As reported, lncRNAs can exhibit diverse functional roles in the process of interactions with proteins, acting as protein decoys to recruit or sequester proteins, or functioning as scaffolds to facilitate the assembly of protein complexes [37, 38]. Our study revealed that lncRNA methylation facilitated the interaction between lncRNA and protein to increase protein stability and further promoted tumorigenesis of GCSCs. In this context, our discoveries provide novel insights into the methylation of lncRNAs, emphasizing the significance of m6A modification in tumorigenesis of GCSCs. It should be noted that the mechanism was validated in three gastric cancer cell lines, including one sample from a patient with gastric cancer and two different typical gastric cancer cell lines. Given the heterogeneity of cancer cells, the conclusions we summarized require further validation with additional samples from patients with gastric cancer. In addition to gastric cancer, the roles of methylated *PSMA3-AS1* and *MIR22HG* in other cancers merits further exploration in future.

## Conclusions

Our findings demonstrated that the methylation level of *PSMA3-AS1* and *MIR22HG*, mediated by METTL3 and METTL14, was significantly elevated in GCSCs. This study revealed that the site-specific m6A modification of *PSMA3-AS1* and *MIR22HG* suppressed apoptosis and promoted the stemness of GCSCs. LncRNA methylation increased the stability of *PSMA3-AS1* and *MIR22HG* to promote proliferation and suppress apoptosis of GCSCs via the *PSMA3-AS1*–miR-411-3p–*AP1G1* or *MIR22HG*–miR-24-3p–*SERTAD1* axis. At the same time, the methylation of *PSMA3-AS1* or *MIR22HG* promoted the interaction between *PSMA3-AS1* and EEF1A1 protein or *MIR22HG* and LRPPRC and stabilized the interacted proteins, leading to the suppression of apoptosis and the promotion of proliferation of GCSCs.

### Supplementary Information

**Additional file 1:** **Table S1.** Potential miRNAs targeted by lncRNAs. **Fig S1.** Detection of the predicted m6A sites of lncRNAs* PSMA3-AS1* **A** and *MIR22HG* **B** in GCSCs using single-base elongation- and ligation-based qPCR amplification analysis. Total RNAs were extracted form GCSCS and then subjected to single-base elongation- and ligation-based qPCR amplification analysis to evaluate the predicted m6A sites on *PSMA3-AS1* (**A**) or *MIR22HG* (**B**). The A232 site with no m6A motif RRACH (R=G/A; H=A/C/U) was used as an input control.

## Data Availability

All data generated or analyzed during this study are included in this published article and its Additional information files.

## Abbreviations

lncRNAs: Long noncoding RNAs
GCSCs: Gastric cancer stem cells
GCNSCs: Gastric cancer non-stem cells
NF-κB: Nuclear factor kappa-light- chain-enhancer of activated B cells
m6A: N6-methyladenosine
METTL3: Methyltransferase-like 3
WTAP: Wilms tumor 1 associated protein
ALKBH5: Alkylation repair homolog protein 5
FTO: Fat-mass and obesity-associated protein
YTHDF1: YTH (YT521-B homology) domain-containing family protein 1
YTHDC1: YTH domain-containing protein 1
IGF2BPs: Insulin-like growth factor 2 mRNA-binding proteins
