# Correction: Methylated lncRNAs suppress apoptosis of gastric cancer stem cells via the lncRNA–miRNA/protein axis

**DOI:** 10.1186/s11658-024-00621-6

**Published:** 2024-07-11

**Authors:** Yuan Ci, Yuan Zhang, Xiaobo Zhang

**Affiliations:** College of Life Sciences, Laboratory for Marine Biology and Biotechnology of Pilot National Laboratory for Marine Science and Technology (Qingdao), Southern Marine Science and Engineering Guangdong Laboratory (Zhuhai), Zhejiang University, Hangzhou, 310058 People’s Republic of China

**Correction:Cellular & Molecular Biology Letters (2024) 29:51** 10.1186/s11658-024-00568-8

Following publication of the original article [1], the authors identified that an incorrect image was used for the control “β-tubulin” of the 4th lane “METTL3-silenced GCSC-HGC MIR22HG” of Fig. 3M.

The incorrected Fig. 3M is:


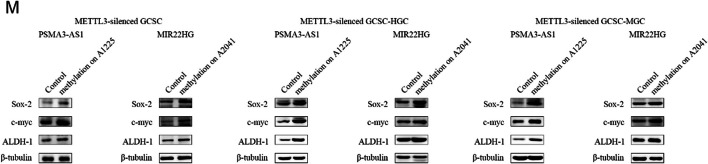


(**M**) Western blot for stemness genes of METTL3-silenced GCSCs with site-specific methylation of lncRNAs.

The corrected Fig. 3M is:


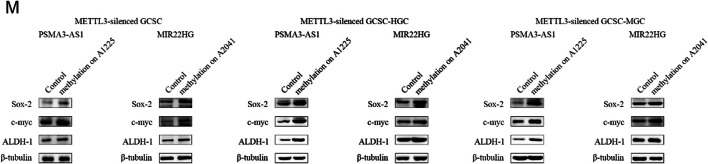


(**M**) Western blot for stemness genes of METTL3-silenced GCSCs with site-specific methylation of lncRNAs.
