# Supplementary material for: Methylated lncRNAs suppress apoptosis of gastric cancer stem cells via the lncRNA–miRNA/protein axis
Source: Cell Mol Biol Lett. 2024 Apr 10;29:51. doi: 10.1186/s11658-024-00568-8 (PMC11005211; doi:10.1186/s11658-024-00568-8)
Supplement: Supplementary file 1 — Additional file 1: Table S1. Potential miRNAs targeted by lncRNAs. Fig S1. Detection of the predicted m6A sites of lncRNAs PSMA3-AS1 A and MIR22HG B in GCSCs using single-base elongation- and ligation-based qPCR amplification analysis. Total RNAs were extracted form GCSCS and then subjected to single-base elongation- and ligation-based qPCR amplification analysis to evaluate the predicted m6A sites on PSMA3-AS1 (A) or MIR22HG (B). The A232 site with no m6A motif RRACH (R=G/A; H=A/C/U) was used as an input control. [file 11658_2024_568_MOESM1_ESM.pdf]

Supplementary materials

Table S1. Potential miRNAs targeted by lncRNAs

lncRNAs	target miRNAs
PSMA3-AS1	miR-101, miR-4504, miR-4429, miR-302a-3p, miR-409-3p, miR-136-5p, miR-378a-3p, miR-376a-3p, miR-411-3p
MIR22HG	hsa-miR-24-3p

Fig S1. Detection of the predicted m6A sites of lncRNAs PSMA3-AS1 (A) and MIR22HG (B) in GCSCs using single-base elongation- and ligation-based qPCR amplification analysis. Total RNAs were extracted form GCSCS and then subjected to single-base elongation- and ligation-based qPCR amplification analysis to evaluate the predicted m6A sites on PSMA3-AS1 (A) or MIR22HG (B). The A232 site with no m6A motif RRACH (R=G/A; H=A/C/U) was used as an input control.

Fig S1

A

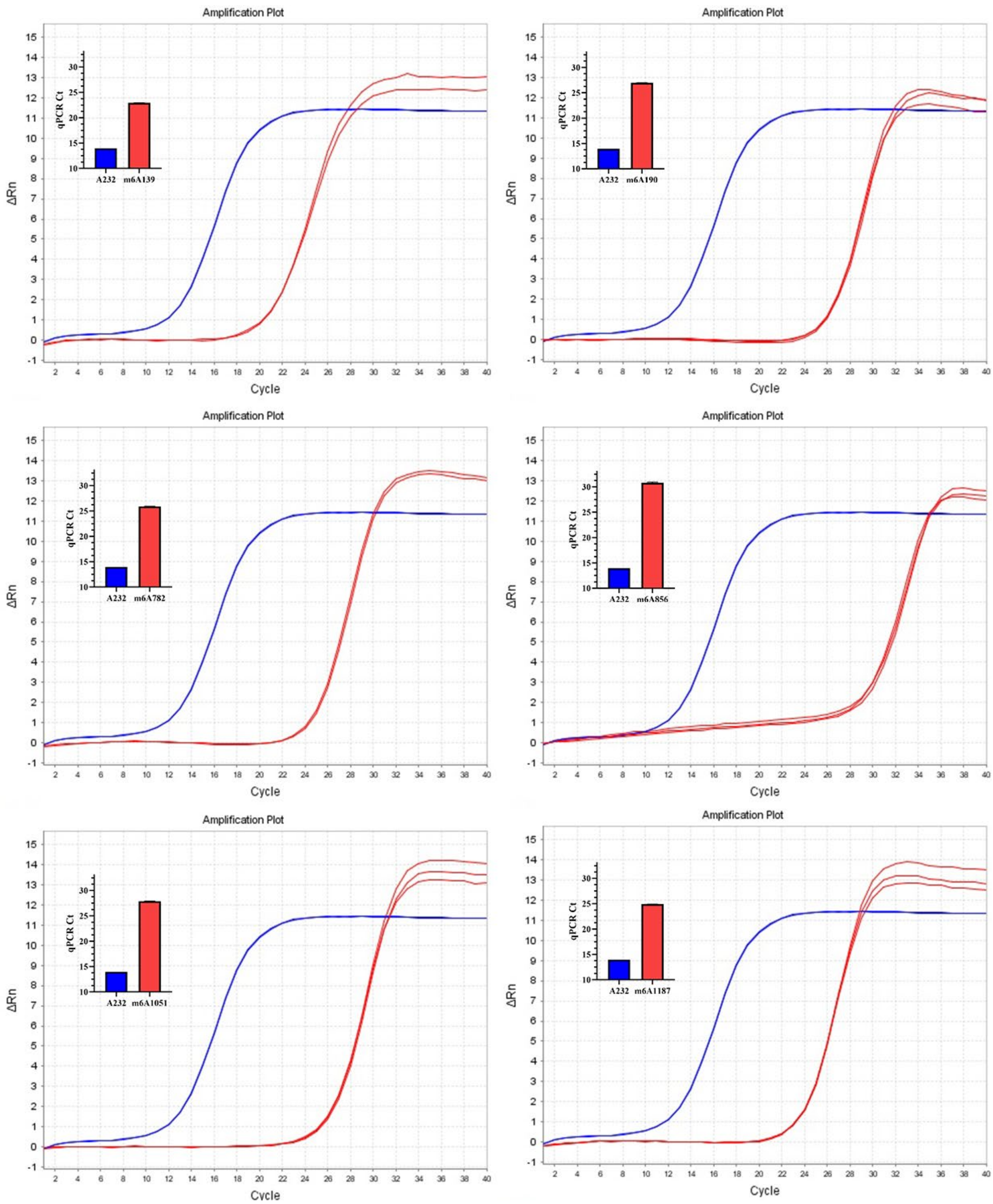
Amplification Plot
ΔRn
Cycle
qPCR Ct
A232
m6A139
m6A190
m6A782
m6A856
m6A1051
m6A1187

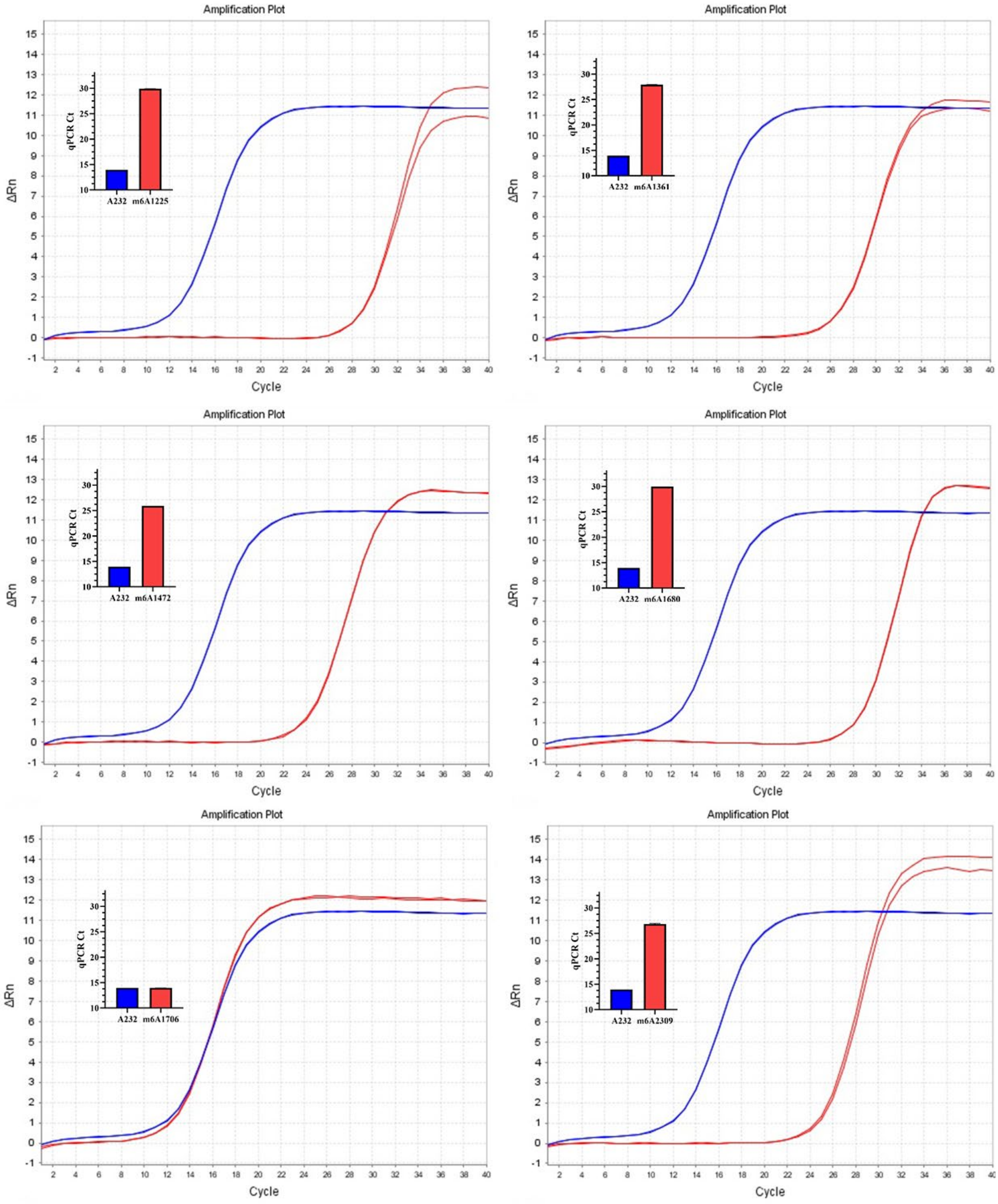
Amplification Plot
ΔRn
Cycle
qPCR Ct
A232
m6A1225
Amplification Plot
ΔRn
Cycle
qPCR Ct
A232
m6A1361
Amplification Plot
ΔRn
Cycle
qPCR Ct
A232
m6A1472
Amplification Plot
ΔRn
Cycle
qPCR Ct
A232
m6A1680
Amplification Plot
ΔRn
Cycle
qPCR Ct
A232
m6A1706
Amplification Plot
ΔRn
Cycle
qPCR Ct
A232
m6A2309

B

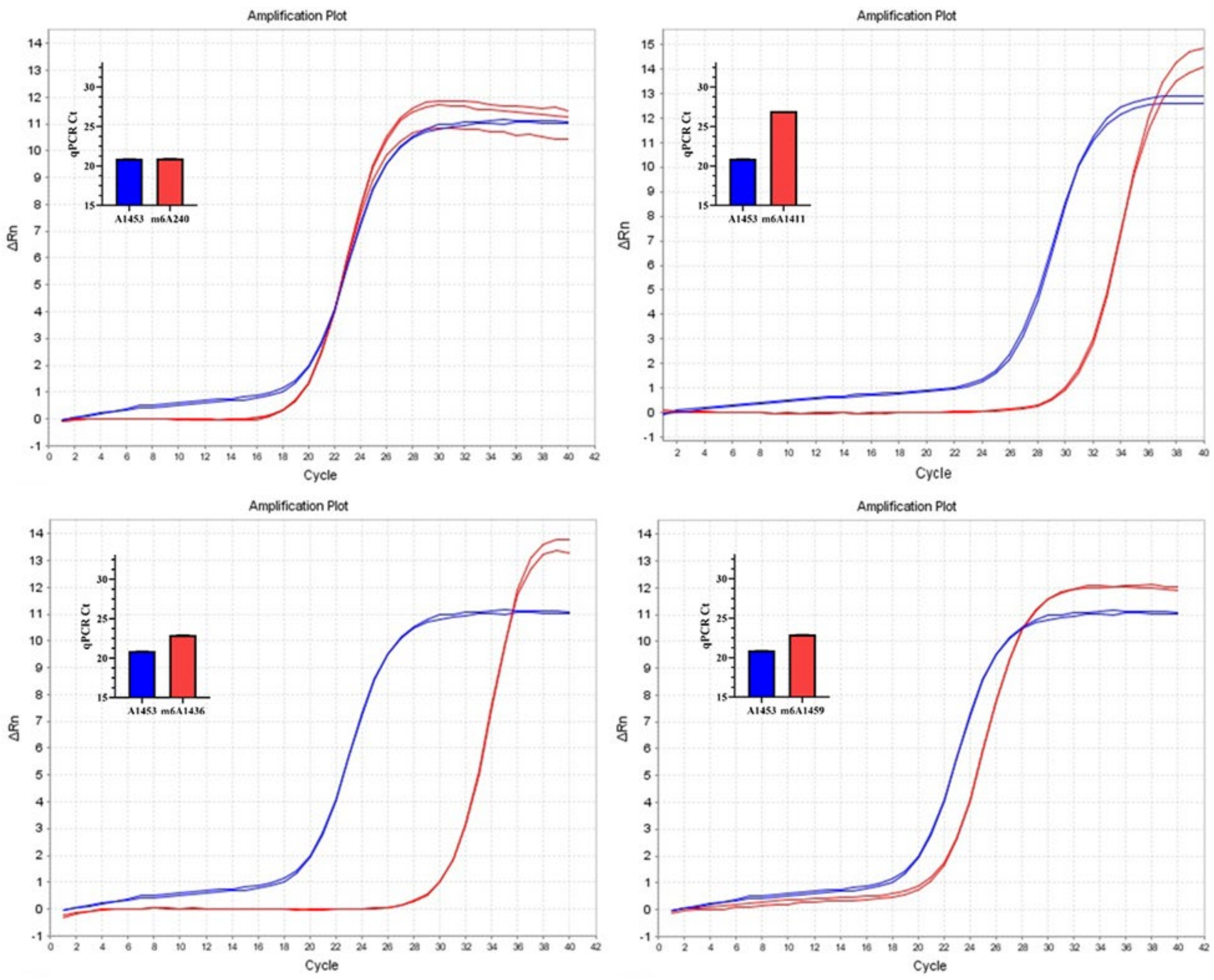

Amplification Plot
ΔRn
Cycle
qPCR Ct
A1453
m6A240
Amplification Plot
ΔRn
Cycle
qPCR Ct
A1453
m6A1411
Amplification Plot
ΔRn
Cycle
qPCR Ct
A1453
m6A1436
Amplification Plot
ΔRn
Cycle
qPCR Ct
A1453
m6A1459

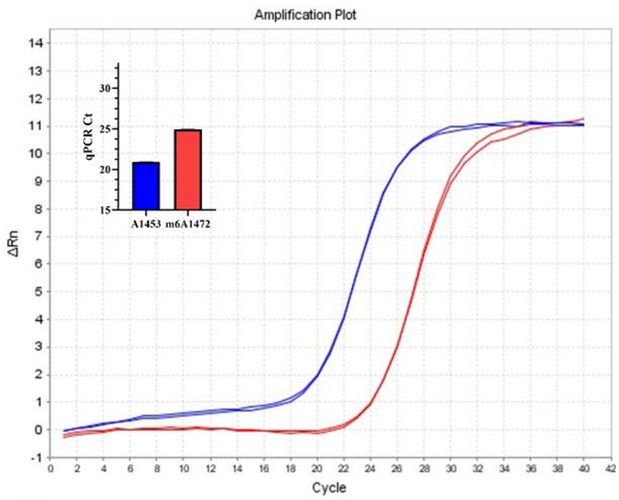
Amplification Plot
qPCR Ct
A1453
m6A1472
ΔRn
Cycle

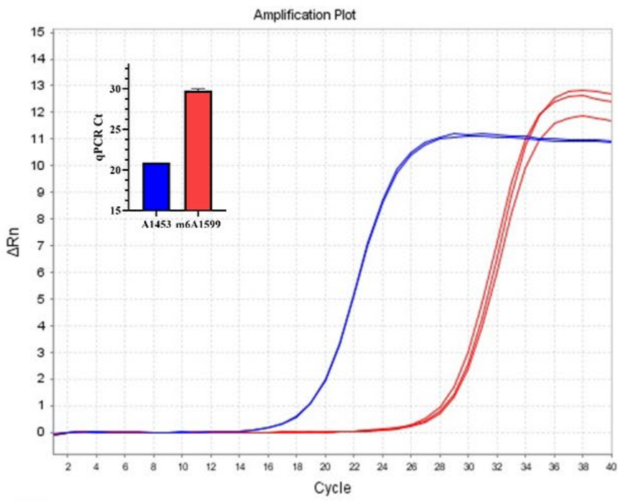
Amplification Plot
qPCR Ct
A1453
m6A1599
ΔRn
Cycle

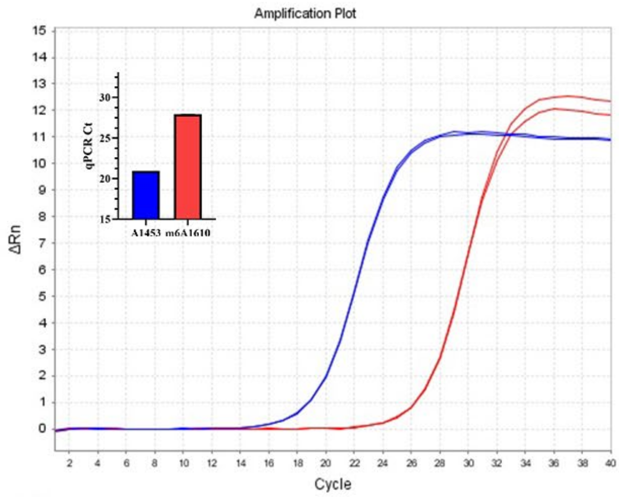
Amplification Plot
qPCR Ct
A1453
m6A1610
ΔRn
Cycle

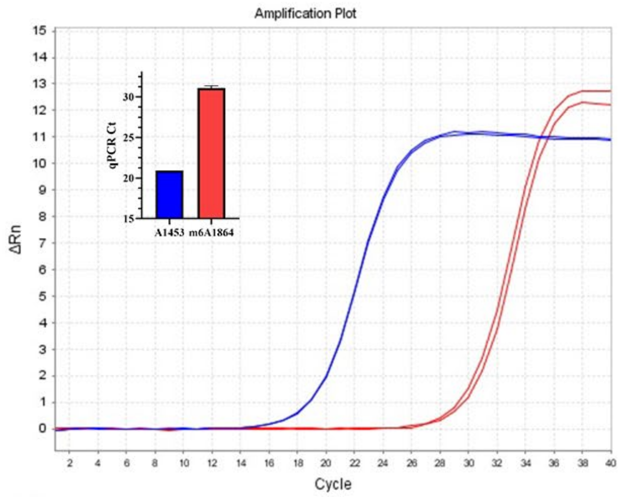
Amplification Plot
qPCR Ct
A1453
m6A1864
ΔRn
Cycle

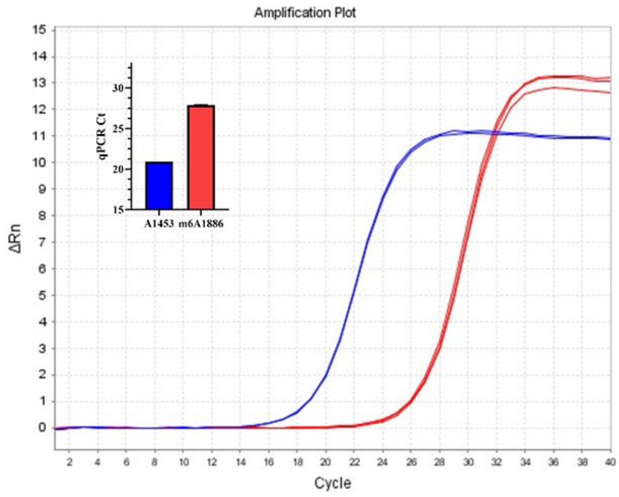
Amplification Plot
qPCR Ct
A1453
m6A1886
ΔRn
Cycle

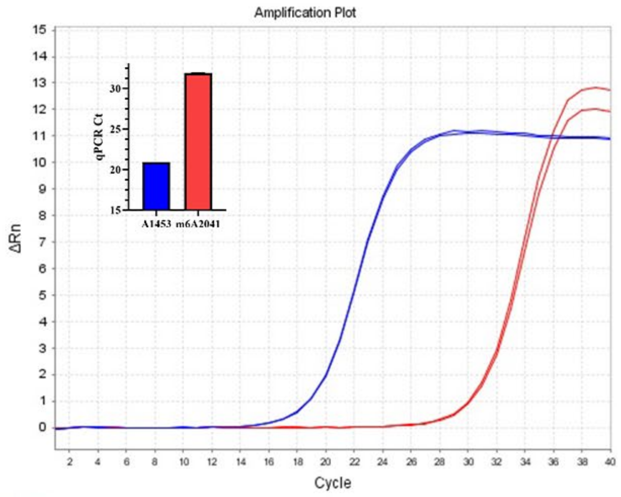
Amplification Plot
qPCR Ct
A1453
m6A2041
ΔRn
Cycle
